# RHOV couples EMT-associated plasticity to cytoskeletal execution of invasion and metastasis

**DOI:** 10.1038/s41420-026-03137-4

**Published:** 2026-05-07

**Authors:** Qi Wang, Yaru Zhao, Shenghui Huang, Haiyan Fu, Chiara Reina, Alexandra Aicher, Jiajia Tang, Christopher Heeschen

**Affiliations:** 1 Center for Single-Cell Omics, School of Public Health, Shanghai Jiao Tong University School of Medicine, Shanghai, China; 2 State Key Laboratory of Systems Medicine for Cancer, Shanghai Jiao Tong University School of Medicine, Shanghai, China; 3 Department of Molecular Biotechnology and Health Sciences, University of Turin, Turin, Italy; 4 Pancreatic Cancer Heterogeneity, Candiolo Cancer Institute FPO-IRCCS, Candiolo, Turin, Italy; 5 Precision Immunotherapy, Graduate Institute of Biomedical Sciences, China Medical University, Taichung, Taiwan

**Keywords:** Pancreatic cancer, Mechanisms of disease

## Abstract

Pancreatic ductal adenocarcinoma (PDAC) is characterized by early invasion and rapid metastatic dissemination, yet the cytoskeletal mechanisms that enable these aggressive behaviors remain incompletely defined. Here, we identify the atypical Rho GTPase RHOV as a critical regulator of invasive progression and metastasis in PDAC. Integrated analyses of independent patient cohorts, patient-derived models, and single-cell transcriptomic datasets revealed that RHOV is selectively overexpressed in malignant epithelial cells, with high RHOV expression correlating with advanced disease stage and poor patient survival. Genetic suppression or deletion of RHOV impaired PDAC cell invasion, migration, clonogenic growth, and context-dependent sphere formation in vitro, while reducing tumor-initiating capacity and metastatic colonization in vivo. Mechanistically, RHOV maintains BRK1-dependent WAVE regulatory complex integrity to sustain lamellipodia formation and invasive motility. Loss of RHOV uncoupled EMT-associated transcriptional programs from cytoskeletal execution of invasion, resulting in compensatory EMT gene expression without restoration of invasive behavior. Re-expression of BRK1 rescued invasion defects following RHOV inhibition. Together, these findings identify RHOV as an executional dependency that enables PDAC invasiveness by linking transcriptional plasticity to actin-based motility.

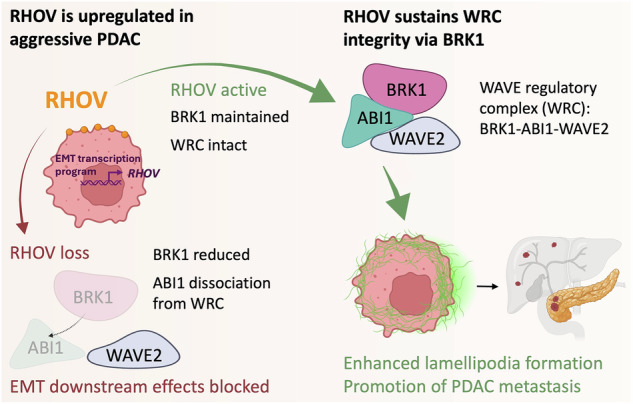

## Background

Pancreatic ductal adenocarcinoma (PDAC) is one of the most lethal human malignancies, with a five-year survival rate below 5% across all stages [1, 2]. Although it accounts for only 2–3% of all cancer diagnoses, PDAC is responsible for nearly 5% of cancer-related deaths worldwide [3]. Most patients present with advanced or metastatic disease, when curative options are no longer feasible, and current treatments – including surgery, chemotherapy, and radiotherapy – provide only limited and transient benefit. These outcomes highlight the need to better understand the molecular mechanisms underlying PDAC aggressiveness and therapy resistance.

A defining feature of PDAC is early local invasion and rapid dissemination, even in tumors that appear anatomically localized. This aggressive behavior reflects pronounced cellular motility and phenotypic plasticity, driven in part by epithelial-mesenchymal transition (EMT), a reversible program that promotes invasion, migration, and resistance to apoptotic stress [4–6]. EMT involves remodeling of cell–cell adhesion and cytoskeletal organization, enabling PDAC cells to adapt their migratory behavior to microenvironmental constraints; however, how EMT-associated transcriptional programs are translated into the execution of invasive behavior remains incompletely understood.

Invasion requires coordinated actin cytoskeletal remodeling to control cell shape, polarity, and movement, with lamellipodia serving as key structures that drive mesenchymal migration through complex tissues. Lamellipodia formation is orchestrated by the WAVE regulatory complex, which couples upstream signaling cues to Arp2/3-mediated actin polymerization [7, 8]. Proper assembly and stability of the WAVE complex are required for sustained protrusive activity and efficient migration [9], yet the molecular mechanisms that maintain WAVE complex function in highly invasive PDAC cells remain poorly defined.

Small GTPases of the Rho family are central regulators of actin dynamics, cell polarity, and migration [4]. While canonical members such as RhoA, Rac1, and Cdc42 have established roles in PDAC invasion and progression [10, 11], atypical Rho GTPases are less well characterized. Among these, RHOV (also known as Chp) is a structurally distinct, fast-cycling GTPase that lacks intrinsic GTP hydrolysis activity and is regulated primarily at the level of expression [12, 13]. RHOV has been implicated in cytoskeletal organization and planar cell polarity in non-malignant systems [4], and elevated RHOV expression has been associated with aggressive behavior in several solid tumors [14]. However, its functional role in PDAC progression and invasive behavior has not been defined.

In this study, we investigate the role of RHOV in regulating cytoskeletal dynamics and invasion in PDAC. We identify RHOV as a regulator of cytoskeletal plasticity and invasive behavior in this disease context. Using genetic loss-of-function approaches in patient-derived PDAC models, we demonstrate that RHOV is required for efficient migration and invasion and for the execution of invasive cytoskeletal programs downstream of EMT-associated transcriptional plasticity. Rather than regulating EMT-associated transcription, RHOV depletion disrupts actin organization and protrusive activity through destabilization of the WAVE regulatory complex. We identify BRK1 as a key downstream effector within this pathway and show that RHOV sustains WAVE complex integrity to enable lamellipodia formation and invasive motility. Together, these findings establish RHOV as an upstream dependency of the WAVE–BRK1 machinery and reveal a previously unrecognized mechanism by which PDAC cells translate transcriptional plasticity into cytoskeletal execution of invasive behavior.

## Results

### RHOV is overexpressed in PDAC and predicts poor outcome

**Fig. 1:**
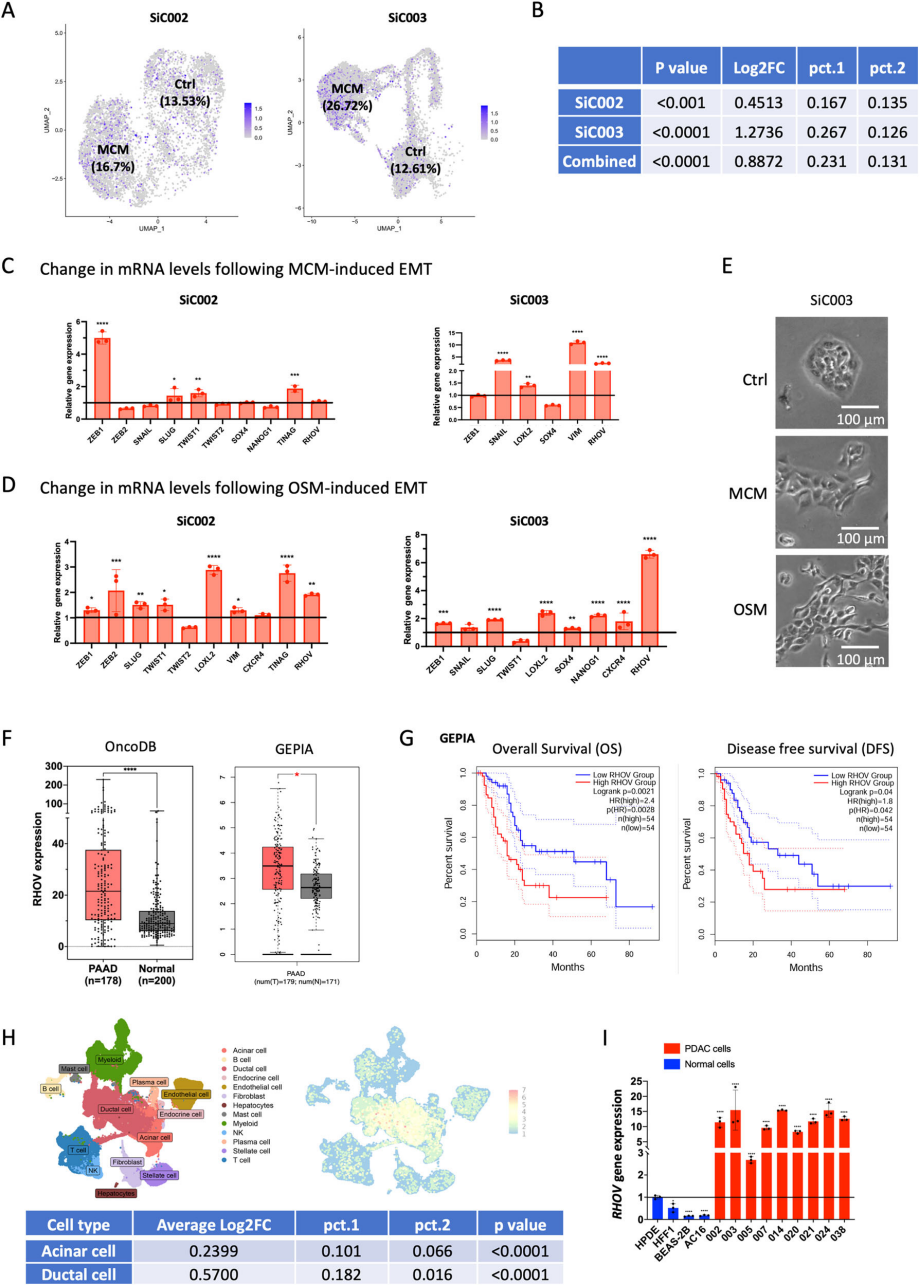
RHOV is overexpressed and predicts poor outcome in PDAC. **A** UMAP visualization of single-cell RNA-seq data from SiC002 and SiC003 under macrophage-conditioned medium (MCM) and control (Ctrl) conditions, with *RHOV* expression overlaid. Percentages indicate the fraction of RHOV-expressing cells within each condition. **B** Differential expression statistics for *RHOV* from single-cell RNA-seq analysis comparing MCM and Ctrl conditions in SiC002, SiC003, and the combined dataset (002 + 003), including *P* value, log2 fold change, and the percentage of *RHOV*-expressing cells in each condition (pct.1 and pct.2). **C** qPCR analysis of a selected panel of EMT transcription factors, EMT effectors, and stemness-associated genes in SiC002 and SiC003 following MCM treatment, shown as relative mRNA levels normalized to control (horizontal line). Data are shown from *n* = 3 independent experiments. **D** qPCR analysis of EMT- and stemness-associated genes in SiC002 and SiC003 following oncostatin M (OSM) treatment, shown as relative mRNA levels normalized to control (horizontal line). Data are shown from *n* = 3 independent experiments. **E** Representative phase-contrast images of SiC003 under Ctrl, MCM, and OSM conditions illustrating cellular morphology. **F** *RHOV* mRNA expression in pancreatic cancer versus normal pancreas using public datasets. Left panel, OncoDB. Right panel, GEPIA. PAAD (PDAC) samples are shown in red and normal tissue in grey. **G** Kaplan–Meier survival analyses (GEPIA) stratified by high and low *RHOV* mRNA expression. Left, overall survival (OS). Right, disease-free survival (DFS). **H** Single-cell RNA-seq atlas of PDAC showing *RHOV* expression across cell types. Left, UMAP with cell-type annotations. Right, UMAP feature plot showing *RHOV* expression intensity. The table summarizes *RHOV* differential expression metrics in acinar and ductal cell populations. **I** *RHOV* mRNA expression measured by qPCR across patient-derived PDAC cultures (SiC002, 003, 005, 007, 020, 021, 024, 038) and non-malignant control cell types (AC16, HFF1, HPDE, and BEAS-2B). Each dot represents an independent qPCR measurement. Statistical significance is indicated as *p < 0.05, **p < 0.01, ***p < 0.001, and ****p < 0.0001. Data are presented as mean ± SD.

Through integrated transcriptomic analyses of multiple public datasets, we identified RHOV as a previously underappreciated candidate gene in pancreatic ductal adenocarcinoma that may be associated with disease progression. To investigate whether *RHOV* expression is dynamically regulated by tumor microenvironmental cues, we analyzed single-cell RNA-seq data from SiC002 and SiC003 PDAC cells cultured under control conditions or macrophage-conditioned medium (MCM), an established EMT-inducing stimulus. UMAP projections revealed a pronounced expansion of *RHOV*-expressing cells following MCM exposure in both models, with a clear increase in the fraction of RHOV-positive cells compared with control conditions (Fig. 1A). Differential expression analysis confirmed significant *RHOV* upregulation in MCM-treated cultures in SiC002, SiC003, and the combined dataset, reflected by increased log2 fold change values and a higher proportion of *RHOV*-expressing cells (Fig. 1B).

To further characterize transcriptional programs associated with *RHOV* regulation, we performed qPCR analyses of *RHOV* together with EMT- and cancer stem cell (CSC)-associated genes following EMT-inducing MCM or oncostatin M (OSM) treatment. MCM exposure induced heterogeneous EMT-associated transcriptional responses in SiC002 and SiC003 cells, with distinct marker induction patterns across models, whereas OSM treatment elicited a more convergent response characterized by robust upregulation of *LOXL2*, accompanied by more modest induction of *SLUG*, together with strong induction of *RHOV*, in both cultures (Fig. 1C, D). CSC-associated gene expression changes were selectively observed under OSM treatment in SiC003 cultures, with increased NANOG1 and CXCR4 expression, indicating context-dependent activation of EMT-associated transcriptional programs. Consistent with these molecular changes, phase-contrast imaging revealed spindle-shaped, mesenchymal-like morphology following both MCM and OSM exposure (Fig. 1E and Fig. S1A).

To assess the clinical relevance of *RHOV* expression, we examined multiple independent patient cohorts. *RHOV* mRNA levels were significantly elevated in pancreatic ductal adenocarcinoma compared with normal pancreas, as shown by analyses from OncoDB and GEPIA (Fig. 1F) and confirmed by density distribution analysis using TCGA and GTEx datasets (Fig. S1B). *RHOV* overexpression was also observed across multiple cancer types, with tumor-specific enrichment relative to matched normal tissues (Fig. S1C), and higher *RHOV* expression was associated with more advanced pathological stages of PDAC (Fig. S1D).

Consistent with these expression patterns, Kaplan-Meier survival analyses demonstrated that elevated *RHOV* expression was significantly associated with reduced overall survival and disease-free survival in PDAC patients (Fig. 1G). This adverse prognostic association was reproducible across multiple independent platforms, including UALCAN, OncoDB, and TIMER2.0 (Fig. S1E).

At the cellular level, analysis of a PDAC single-cell RNA-seq atlas revealed that *RHOV* expression was preferentially enriched within malignant epithelial compartments, with notably higher expression in ductal-like tumor cells (Fig. 1H). Finally, qPCR analysis across a panel of patient-derived PDAC cultures confirmed consistently elevated *RHOV* expression relative to non-malignant control cell types (Fig. 1I).

Collectively, these data identify RHOV as a microenvironment-responsive gene that is preferentially expressed in malignant PDAC epithelium and associated with EMT-linked states, advanced disease, and poor clinical outcome.

### RHOV promotes aggressiveness of human PDAC cells

Given that RHOV is upregulated following EMT induction by MCM and OSM, we next asked whether RHOV is required to translate EMT-associated transcriptional programs into the functional and cytoskeletal execution of invasion. EMT encompasses multiple separable layers including transcriptional activation, structural remodeling of cell–cell junctions, and cytoskeletal execution of invasive motility. In the following analyses, we explicitly distinguish EMT-associated transcriptional activation from structural and functional execution of invasion. This distinction allows us to specifically interrogate whether RHOV is required for EMT transcription per se or acts downstream as a cytoskeletal dependency enabling invasive behavior.

**Fig. 2:**
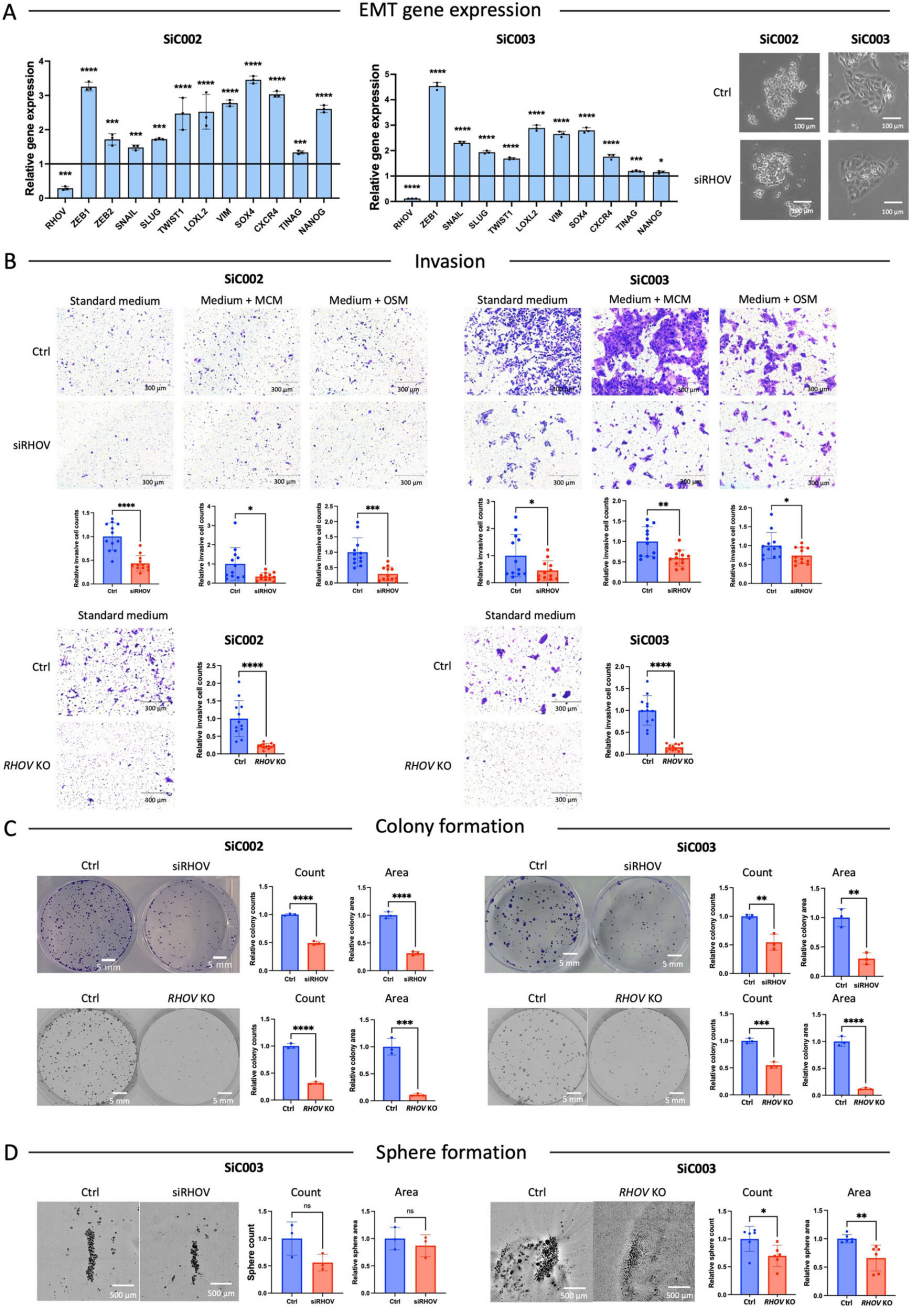
RHOV promotes aggressiveness of human PDAC cells. **A** qPCR analysis of EMT-associated gene expression in SiC002 (left) and SiC003 (middle) cells following *RHOV* knockdown. Genes include EMT transcription factors, EMT effectors, stemness-associated markers, and ECM-related EMT markers. Data are based on *n* = 3 independent experiments per condition. Representative phase-contrast images of control and *RHOV*-depleted SiC002 and SiC003 cells are shown (right). **B** Transwell invasion assays of SiC002 and SiC003 primary PDAC cultures following *RHOV* knockdown by siRHOV under standard medium, MCM, or OSM conditions. Invasion assays of *RHOV* knockout single-cell clones derived from SiC002 and SiC003 are shown below. Representative images are shown together with quantification of relative invasive cell counts. Each dot represents one independently imaged field. **C** Colony formation assays of SiC002 and SiC003 cells following *RHOV* knockdown by siRNA (upper panels) or *RHOV* knockout (lower panels). Representative colony images are shown together with quantification of colony number and colony area. Each dot represents one independently analyzed well. **D** Sphere formation assays of SiC003 cells following *RHOV* knockdown by siRNA (left) or *RHOV* knockout (right). Representative images are shown together with quantification of sphere number and sphere area. Each dot represents one independently analyzed culture well. Data are presented as mean ± SD. Statistical significance is indicated as *p < 0.05, **p < 0.01, ***p < 0.001, and ****p < 0.0001.

To first explore the relationship between RHOV and EMT-associated transcriptional programs, we performed qPCR analyses following *RHOV* knockdown. Despite robust induction of EMT-associated transcription factors, RHOV-depleted cells retained epithelial-like morphology (Fig. 2A), indicating a compensatory EMT-associated transcriptional response without corresponding structural EMT execution. Specifically, although EMT-associated genes such as *ZEB1, SNAI1, SLUG, TWIST1, LOXL2*, and *VIM* were upregulated following RHOV knockdown in both SiC002 and SiC003 cells, RHOV-deficient cells maintained a compact epithelial-like morphology rather than undergoing characteristic EMT-associated morphological changes. Together, these observations indicate that EMT-associated transcriptional activation alone is insufficient to induce morphological EMT or invasive behavior in the absence of RHOV.

To define the functional role of *RHOV* in human PDAC cells, we performed loss-of-function studies using transient siRNA-mediated knockdown, stable shRNA-mediated suppression, and CRISPR–Cas9–mediated gene knockout across multiple patient-derived PDAC cultures. Efficient reduction of *RHOV* mRNA expression was confirmed by qPCR under standard culture conditions as well as following MCM or OSM exposure (Fig. S2A).

To systematically evaluate the pleiotropic role of RHOV in PDAC malignancy, we first examined effects on invasive behavior, followed by analyses of cytoskeletal mechanisms and context-dependent growth phenotypes. To determine whether RHOV loss impairs basic cellular fitness independently of its role in invasion, we assessed cell proliferation. RHOV knockdown in SiC002 and SiC003 cells reduced proliferation, with statistically significant differences observed only after completion of the invasion assay window. A comparable reduction in proliferation was observed in RHOV knockout clones (Fig. S2B). Importantly, invasion assays were completed within a time window preceding statistically significant divergence of the proliferation curves (24–48 hours). We next examined the impact of *RHOV* loss on invasive behavior. In Transwell invasion assays, siRNA-mediated *RHOV* knockdown significantly reduced invasion in both SiC002 and SiC003 under standard culture conditions as well as following MCM or OSM exposure (Fig. 2B). This phenotype was further accentuated in *RHOV* knockout single-cell clones derived from both models (Fig. 2B), and extended across multiple patient-derived PDAC cultures (SiC005, SiC007, SiC610) and an independent knockout clone of SiC005 (Fig. S2C), supporting the generalizability of this effect.

Consistent with defective invasion, *RHOV* suppression also impaired migratory capacity. Wound-healing assays showed delayed gap closure following siRNA- or shRNA-mediated *RHOV* knockdown, as well as in *RHOV* knockout cells, in both SiC002 and SiC003 (Fig. S2D). Notably, this occurred despite the concurrent upregulation of EMT-associated transcripts, demonstrating that *RHOV* is dispensable for EMT-associated transcriptional activation but is required for efficient cytoskeletal execution of invasive behavior.

To assess long-term proliferative and survival capacity, we next performed clonogenic growth assays. *RHOV* knockdown significantly reduced both colony number and colony area in SiC002 and SiC003 (Fig. 2C), with genetic ablation producing an even more pronounced suppression (Fig. 2C and Fig. S2E), reinforcing the role of RHOV in sustaining long-term fitness.

Finally, to evaluate context-dependent effects of RHOV loss on self-renewal-associated growth, we performed sphere formation assays. Under standard culture conditions, siRNA-mediated *RHOV* knockdown did not significantly affect sphere number or size in either SiC002 or SiC003 (Fig. 2D and Fig. S2F). Strikingly, sphere formation defects emerged upon OSM exposure in SiC002 following *RHOV* knockdown, suggesting that partial RHOV loss sensitizes cells to inflammatory stress (Fig. S2F). In contrast, RHOV knockout cells exhibited pronounced sphere formation defects even under basal conditions (Fig. 2D), revealing a more stringent requirement for RHOV when completely absent. These findings demonstrate a dose-dependent requirement for RHOV in self-renewal: partial loss confers context-dependent defects under inflammatory stress, while complete loss exposes an essential role in basal sphere formation. In addition, *RHOV* knockdown increased in vitro sensitivity to chemotherapeutic agents in PDAC cells, as combined siRHOV and drug treatment resulted in greater reductions in cell viability compared with drug treatment alone across multiple conditions (Fig. S2G).

Collectively, these data demonstrate that *RHOV* is required to couple EMT-associated transcriptional plasticity to cytoskeletal execution of invasion and migration, while supporting context-dependent clonogenic and sphere-forming capacity and modulating in vitro chemosensitivity in PDAC cells.

### RHOV promotes PDAC initiation and progression in vivo

**Fig. 3:**
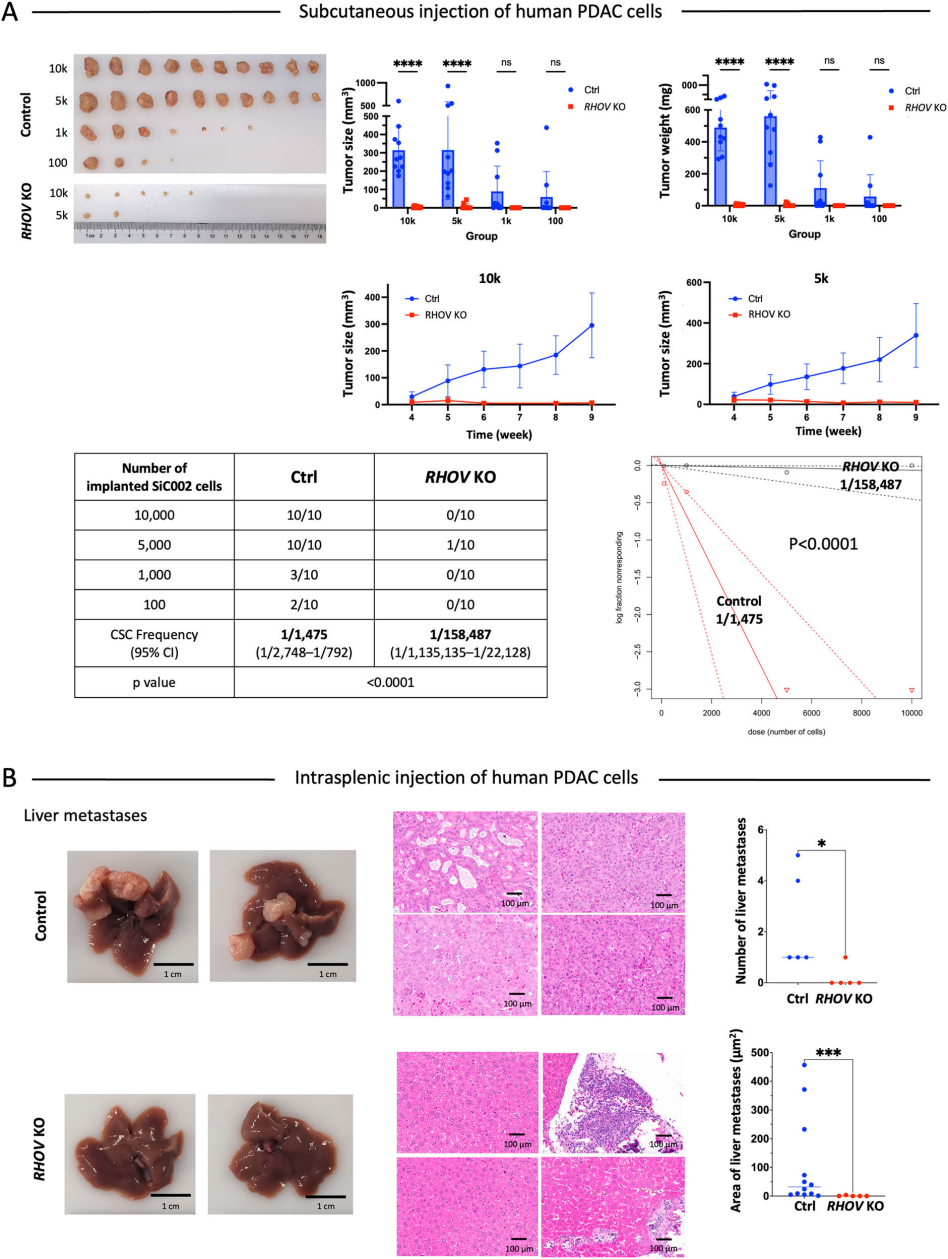
RHOV enhances tumor initiation and metastasis in vivo. **A** Subcutaneous tumor formation following injection of human PDAC cells (SiC002) with control or *RHOV* knockout (KO). Top left, representative images of excised tumors generated from decreasing numbers of injected cells (10,000, 5,000, 1,000, and 100 cells; k = thousand). For each condition and cell dose, *n* = 10 mice were analyzed. Top middle and right, quantification of tumor size and tumor weight for each injection dose. Bottom, tumor growth curves over time for tumors generated from 10,000 or 5,000 injected cells (*n* = 10 mice per group). The table summarizes tumor incidence across limiting dilution groups, and the adjacent plot depicts extreme limiting dilution analysis (ELDA: https://bioinf.wehi.edu.au/software/elda/) used to estimate CSC frequency. **B** Liver metastasis following intrasplenic injection of control or *RHOV* knockout SiC002 cells. Left, representative macroscopic images of livers from control and *RHOV* knockout groups. Middle, representative H&E-stained liver sections. Right, quantification of liver metastatic burden shown as the number of metastatic lesions and total metastatic area (*n* = 10 mice per group). Data are presented as mean ± SEM. Statistical significance is indicated as *p < 0.05, ***p < 0.001, ****p < 0.0001.

To assess the role of *RHOV* in tumor initiation and metastatic progression in vivo, we performed subcutaneous and intrasplenic transplantation assays using human PDAC cells. In limiting dilution experiments, *RHOV* knockout resulted in near-complete suppression of tumor outgrowth across all tested cell doses, indicating a cell-intrinsic requirement for RHOV in sustaining in vivo tumor growth and initiation, rather than merely modulating growth kinetics (Fig. 3A). At higher inoculation doses (10,000 and 5,000 cells), tumors derived from *RHOV* knockout cells failed to expand over time, whereas control tumors exhibited progressive growth. This growth defect was accompanied by pronounced alterations in tumor histology, with H&E staining revealing disrupted tumor architecture in *RHOV* knockout tumors compared with controls (Fig. S3A).

At lower inoculation doses (1,000 and 100 cells), tumor formation was largely absent in the *RHOV* knockout group, indicating a profound loss of tumor-initiating capacity. Extreme limiting dilution analysis demonstrated a marked reduction in CSC frequency upon *RHOV* deletion. Whereas control SiC002 cells exhibited a CSC frequency of 1 in 1,475 cells, *RHOV* knockout cells displayed a CSC frequency of 1 in 158,487 cells, corresponding to an approximately 100-fold reduction in tumor-initiating capacity (Fig. 3A). Immunohistochemical analyses of subcutaneous tumors supported these findings, revealing reduced Ki-67 positivity in *RHOV* knockout tumors, consistent with diminished proliferative activity in vivo (Fig. S3B). PanCK and CD45 staining confirmed tumor epithelial identity and delineated immune cell infiltration, respectively (Fig. S3B).

To determine whether RHOV also contributes to metastatic progression, we next performed intrasplenic injections to assess liver colonization. Control cells consistently formed macroscopically visible liver metastases, whereas *RHOV* knockout cells exhibited a pronounced reduction in metastatic burden (Fig. 3B). Quantitative analysis demonstrated significant decreases in both the number of liver metastatic lesions and the total metastatic area in the *RHOV* knockout group. These observations were corroborated at the histological level by H&E staining of liver sections, which revealed markedly reduced metastatic involvement in animals injected with *RHOV* knockout cells (Fig. 3B and Fig. S3C). Immunohistochemistry for Ki67 and PanCK confirmed liver metastases in control animals. In contrast, liver sections from animals injected with *RHOV* knockout cells lacked detectable metastatic lesions, with Ki-67 and PanCK staining patterns consistent with normal liver parenchyma, precluding quantitative comparison of intralesional marker expression (Fig. S3C). In contrast, lung metastatic burden was not significantly altered, as assessed by H&E staining, quantification of lung metastases, and immunohistochemistry for Ki67, PanCK, CD45, and RHOV (Fig. S3D, E). This site-specific effect is consistent with a requirement for RHOV in colonization efficiency rather than generalized survival or dissemination across all metastatic niches. More detailed immune infiltration analyses were limited by the use of immunocompromised mouse models.

Together, these in vivo data establish RHOV as a critical determinant of tumor-initiating capacity, sustained tumor growth, and metastatic colonization in PDAC, while acknowledging that these models evaluate tumor cell growth and organ colonization capacity, rather than the complete PDAC metastatic cascade.

### RHOV regulates lamellipodia formation through the WAVE regulatory complex

**Fig. 4:**
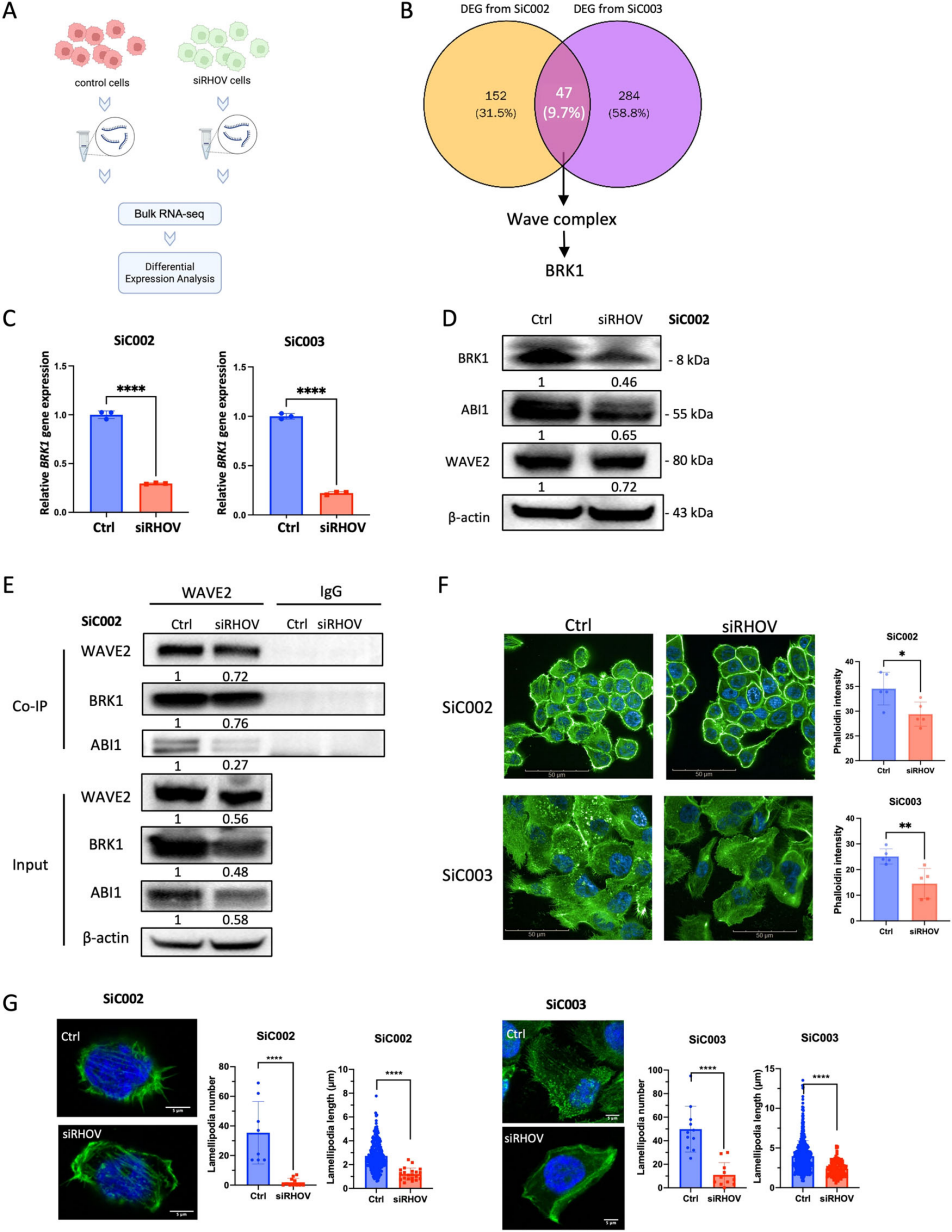
RHOV regulates lamellipodia formation through the WAVE regulatory complex. **A** Schematic overview of the bulk RNA-sequencing workflow used to identify genes regulated by *RHOV* knockdown in SiC002 and SiC003 PDAC cultures. **B** Venn diagram showing the total number of differentially expressed genes (DEGs) identified following RHOV knockdown in SiC002 and SiC003 cells, as well as the overlapping DEGs shared between both datasets. **C** qPCR analysis of *BRK1* mRNA expression following *RHOV* knockdown in SiC002 and SiC003 cells (*n* = 3 independent experiments per condition). **D** Western blot analysis of WAVE regulatory complex–associated proteins in SiC002 cells following *RHOV* knockdown. Representative blots for BRK1, ABI1, and WAVE2 are shown, with β-actin as a loading control. Band intensities were quantified by densitometry and normalized to β-actin. Values shown below blots represent relative protein abundance. **E** Co-immunoprecipitation analysis of the WAVE regulatory complex in SiC002 cells following *RHOV* knockdown. WAVE2 was immunoprecipitated, and associated *BRK1* and *ABI1* were detected; IgG served as a negative control, and input lysates are shown. **F** Phalloidin staining of filamentous actin in SiC002 and SiC003 cells following *RHOV* knockdown. Representative images are shown together with quantification of phalloidin fluorescence intensity, calculated as the mean of multiple microscopic fields per experiment and averaged across *n* = 3 independent experiments. **G** Representative images and quantification of lamellipodia number and lamellipodia length following *RHOV* knockdown in SiC002 and SiC003 cultures (each dot represents one independently imaged microscopic field). Statistical significance is indicated as *p < 0.05, **p < 0.01, and ****p < 0.0001. Data are presented as mean ± SD.

To define the molecular mechanisms by which RHOV regulates cytoskeletal execution of invasive behavior, we performed bulk RNA sequencing following *RHOV* knockdown in two independent patient-derived PDAC cultures, SiC002 and SiC003 (Fig. 4A and Fig. S4A). Differential expression analysis identified a reproducible set of genes regulated upon *RHOV* depletion in both models, with 47 overlapping differentially expressed genes (DEGs) shared between SiC002 and SiC003 (Fig. 4B and Fig. S4B). Among these *RHOV*-regulated genes, components associated with the WAVE regulatory complex emerged as the most consistently enriched cytoskeletal dependency, suggesting a direct link between RHOV and lamellipodia control.

Gene Ontology cellular component enrichment analysis highlighted the SCAR complex, which corresponds to the WAVE regulatory complex, providing a molecular framework for RHOV-dependent regulation of actin-based protrusive structures. Additional enriched cellular components included muscle thin filament tropomyosin and bicellular tight junctions, consistent with alterations in actin-associated structures and cell–cell interfaces. Beyond cytoskeletal terms, the shared *RHOV*-regulated gene set was also enriched for pathways related to cell cycle control, vesicle trafficking, and DNA repair, indicating broader transcriptional changes accompanying RHOV-dependent cytoskeletal perturbation (Fig. S4C).

Within the shared DEG set, *BRK1* – a core structural component of the WAVE regulatory complex – emerged as a candidate effector linking RHOV to actin remodeling. While BRK1 was the only WAVE regulatory complex component present within the shared 47-gene DEG set, we additionally examined other core complex members, including ABI1 and CYFIP1, given their essential structural roles within the WAVE regulatory complex (Fig. S4D). qPCR analysis confirmed reduced *BRK1* mRNA expression following siRNA-mediated *RHOV* knockdown in both PDAC models (Fig. 4C).

Consistent with these RNA-sequencing data, BRK1 exhibited strong and concordant downregulation in both SiC002 and SiC003 (log2 fold change −2.06 and −2.22, respectively; FDR < 0.0001). In contrast, RNA-sequencing revealed modest but reproducible upregulation of selected WAVE regulatory complex–associated genes, including ABI1 (log2 fold change 0.22 in both models) and CYFIP1 (log2 fold change 0.18 and 0.10), consistent with the direction of change observed by qPCR (Fig. S4D). However, the magnitude of these increases was substantially smaller than the loss of BRK1 and was insufficient to functionally compensate for *BRK1* depletion or restore WAVE regulatory complex integrity, as demonstrated by protein expression and complex assembly analyses. These findings support a compensatory transcriptional response rather than functional rescue following RHOV loss. To determine whether these transcriptional changes were reflected at the protein level, we performed Western blot analyses in SiC002 cells. Consistent with the RNA-seq data, BRK1 protein levels were strongly reduced upon *RHOV* knockdown, whereas ABI1 showed a modest decrease and WAVE2 protein levels remained largely unchanged (Fig. 4D). This pattern indicates selective destabilization of specific WAVE regulatory complex components rather than uniform complex degradation. To directly assess whether *RHOV* depletion affects WAVE regulatory complex assembly, we performed co-immunoprecipitation analyses using WAVE2 as bait. *RHOV* knockdown resulted in a pronounced loss of ABI1 association with WAVE2, whereas BRK1 binding to WAVE2 was largely preserved, and WAVE2 itself showed no substantial change in recovery, indicating partial disassembly and functional destabilization of the WAVE regulatory complex rather than complete complex collapse (Fig. 4E). At the cellular level, phalloidin staining revealed altered filamentous actin organization following *RHOV* depletion in both SiC002 and SiC003 cultures, accompanied by reduced phalloidin fluorescence intensity (Fig. 4F). Consistent with these cytoskeletal alterations, quantitative analysis demonstrated a significant reduction in both lamellipodia number and lamellipodia length per cell upon *RHOV* loss (Fig. 4G), supporting a direct role for RHOV in sustaining lamellipodia formation through WAVE–BRK1–dependent actin remodeling.

Thus, *RHOV* loss perturbs WAVE regulatory complex integrity and lamellipodia formation, characterized by strong BRK1 downregulation at the protein level and impaired ABI1 incorporation into the complex, culminating in defective lamellipodia formation despite compensatory transcriptional responses, thereby identifying RHOV as a regulator of invasive actin remodeling rather than general actin dynamics.

### RHOV drives PDAC invasiveness through BRK1

To determine whether BRK1 functions as a downstream effector of RHOV in PDAC, we first assessed BRK1 expression and manipulability across PDAC models. Efficient siRNA-mediated *BRK1* knockdown was confirmed in both SiC002 and SiC003 cells (Fig. S5A), enabling subsequent functional analyses. Importantly, *RHOV* mRNA expression was not altered upon *BRK1* knockdown (Fig. S5B), supporting a predominantly unidirectional regulatory relationship in which RHOV acts upstream of BRK1.

**Fig. 5:**
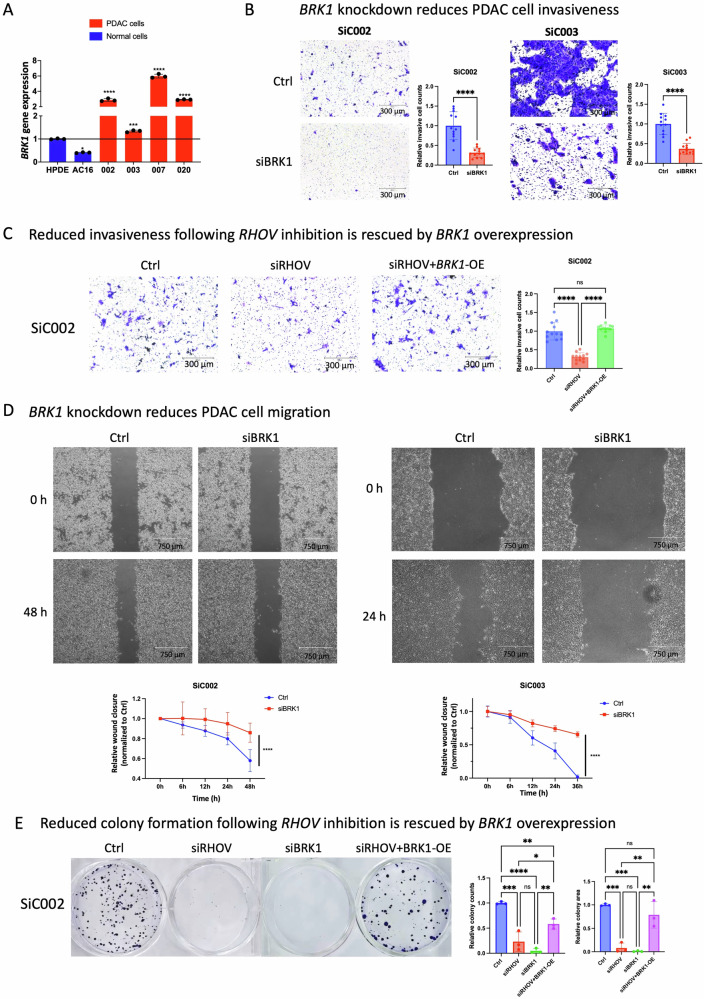
RHOV drives PDAC invasiveness through *BRK1.* **A** qPCR analysis of *BRK1* mRNA expression in patient-derived PDAC cells compared with non-malignant control cells (each dot represents one independent qPCR measurement for the indicated cell culture). **B** Transwell invasion assays in SiC002 and SiC003 cells following *BRK1* knockdown by siRNA. Representative images and quantification of invasive cells are shown (each dot represents one independently imaged transwell insert). **C** Transwell invasion assays assessing invasive capacity following *RHOV* knockdown with or without *BRK1* overexpression in SiC002 cells, with representative images and quantification (each dot represents one independently imaged field). **D** Wound-healing assays evaluating migratory capacity after *BRK1* knockdown by siRNA in SiC002 and SiC003 cells. Representative images at the indicated time points and quantification of wound closure over time are shown (*n* = 5 independently analyzed wounds per time point). **E** Colony formation assays of SiC002 cells following *RHOV* or *BRK1* knockdown, with or without *BRK1* overexpression. Representative colony images are shown together with quantification of colony number and colony area. Each dot represents one independently analyzed well. Statistical significance is denoted as *p < 0.05, ***p < 0.001, and ****p < 0.0001.

Consistent with a potential role in PDAC-associated invasive behavior, qPCR analysis revealed elevated *BRK1* mRNA expression in PDAC cells compared with non-malignant control cells (Fig. 5A).

To directly evaluate the contribution of BRK1 to tumor cell invasiveness, Transwell invasion assays were performed following *BRK1* knockdown in SiC002 and SiC003 cells. Silencing of *BRK1* resulted in a marked reduction in invasive capacity compared with control cells (Fig. 5B). Importantly, differences in cell proliferation following *RHOV* or *BRK1* perturbation emerged only after completion of the migration and invasion assay time window (Figs. S2B and S5C), effectively excluding altered proliferation as a confounding factor in these assays.

To test whether BRK1 mediates RHOV-dependent invasion, we next performed invasion assays following *RHOV* inhibition in the presence or absence of *BRK1* overexpression. Restoration of *BRK1* expression significantly rescued the invasion defect induced by *RHOV* knockdown (Fig. 5C), indicating that BRK1 acts as a functional downstream mediator linking RHOV to invasive behavior. qPCR analysis confirmed strong *BRK1* overexpression in the rescue condition, exceeding baseline levels, while *RHOV* mRNA levels remained suppressed under siRHOV conditions despite *BRK1* overexpression (Fig. S5D).

In addition to invasion, we examined the role of BRK1 in PDAC cell migration using wound-healing assays. *BRK1* silencing impaired wound closure over time in both SiC002 and SiC003 cells, consistent with reduced migratory capacity (Fig. 5D). To further exclude indirect effects due to altered cell growth, proliferation was monitored following *BRK1* knockdown. A measurable reduction in cell proliferation was observed only at later time points (Fig. S5C), confirming that the observed defects in invasion and migration precede detectable changes in cell growth. To assess long-term proliferation and self-renewal capacity, we further conducted colony formation assays using SiC002 cells. A decrease in both the number and size of colonies was observed in both the *BRK1* knockdown and the *RHOV* knockdown groups (Fig. 5E). However, overexpression of *BRK1* partially rescued the inhibitory effect caused by *RHOV* knockdown (Fig. 5E), indicating that the role of RHOV in maintaining long-term proliferative and survival capacity is mediated through BRK1.

Together, these findings establish BRK1 as a downstream effector that mediates RHOV-dependent invasion and migration in PDAC cells.

## Discussion

Although EMT-associated transcriptional programs are strongly linked to PDAC aggressiveness, the cytoskeletal mechanisms that translate this plasticity into effective invasive behavior remain incompletely understood. In this study, we identify the atypical Rho GTPase RHOV as a previously underappreciated regulator of cytoskeletal plasticity and invasive behavior in PDAC. Through integrated analyses of patient cohorts, patient-derived cellular models, in vivo tumorigenicity assays and intrasplenic metastasis assays, we demonstrate that RHOV is consistently overexpressed in PDAC, enriched within malignant epithelial compartments, and associated with advanced disease and poor clinical outcome. Functional perturbation of RHOV reveals a fundamental requirement for this GTPase in sustaining invasion, migration, clonogenic growth, tumor initiation, and metastatic colonization. Mechanistically, our data uncover a previously unrecognized dependency of PDAC cells on RHOV to maintain WAVE regulatory complex integrity, with BRK1 emerging as a key downstream effector that links RHOV activity to lamellipodia formation and invasive motility.

### Pathophysiological context

Rho family GTPases are central regulators of cytoskeletal actin dynamics, cell polarity, and migration. Canonical members such as RhoA, Rac1, and Cdc42 have been extensively studied in PDAC progression and metastasis. In contrast, atypical Rho GTPases remain poorly understood in cancer. RHOV, also known as Chp, belongs to a distinct subgroup characterized by constitutive activity and regulation primarily at the level of expression rather than classical GDP–GTP cycling. RHOV was originally identified as a Cdc42 homolog capable of reorganizing the actin cytoskeleton and activating downstream stress signaling pathways [16]. While RHOV has been implicated in cytoskeletal organization and planar polarity in non-malignant developmental systems, its functional role in PDAC has not been defined. Our findings position RHOV as a cell-intrinsic contributor to invasive and tumor-propagating behavior in PDAC, thereby expanding the repertoire of Rho GTPases implicated in pancreatic cancer progression. Consistent with this concept, unbiased CRISPR-based in vivo screening has recently identified RHOV as a driver of metastatic competence in breast cancer, supporting a broader role for RHOV in tumor dissemination across epithelial malignancies [17].

A key insight from this study is that EMT-associated transcriptional activation alone is insufficient to drive invasion in PDAC cells. Although RHOV depletion triggered compensatory upregulation of EMT-related transcripts, this response did not restore invasive capacity or induce mesenchymal morphology. Instead, RHOV-deficient cells retained epithelial architecture and displayed impaired invasive behavior despite activation of EMT-associated transcriptional programs. These observations indicate that EMT-associated transcriptional plasticity is not sufficient to generate invasive motility in the absence of intact cytoskeletal machinery. In this context, RHOV functions not as a regulator of EMT transcription itself but as a critical determinant of cytoskeletal dynamics required for invasive behavior.

At the mechanistic level, integrated transcriptomic and functional analyses converge on the WAVE regulatory complex as the principal cytoskeletal effector downstream of RHOV. Gene ontology enrichment, protein-level analysis of WAVE regulatory complex components including BRK1, and phalloidin-based imaging of filamentous actin organization consistently revealed disruption of lamellipodia formation following RHOV loss. This mechanism aligns with established models in which the WAVE regulatory complex serves as a central integrator of upstream Rho-family signals to drive Arp2/3-dependent lamellipodia formation [8, 18]. Among WAVE complex components, BRK1 emerged as uniquely sensitive to RHOV depletion at both the transcript and protein levels. Although ABI1 protein levels were only modestly affected, its association with WAVE2 was strongly reduced upon *RHOV* knockdown, indicating impaired WAVE regulatory complex assembly rather than direct ABI1 downregulation. Functional rescue experiments demonstrated that re-expression of BRK1 restored the invasion defect caused by *RHOV* inhibition, supporting a model in which RHOV-dependent regulation of BRK1 contributes to invasive actin remodeling via the WAVE complex.

Importantly, the requirement for RHOV extended beyond in vitro invasion assays. Genetic ablation of *RHOV* profoundly impaired tumor initiation and growth in vivo, with limiting dilution analyses revealing an approximately 100-fold reduction in tumor-initiating capacity. *RHOV* knockout tumors exhibited disrupted architecture, reduced proliferative activity, and diminished liver colonization following intrasplenic injection. These findings indicate that RHOV-dependent cytoskeletal regulation supports tumor growth and organ colonization following direct implantation, rather than establishing a role in the full spontaneous metastatic cascade of PDAC.

### Potential therapeutic implications

In addition to its role in invasion and tumor initiation, RHOV loss enhanced sensitivity to chemotherapeutic agents commonly used in PDAC treatment, including gemcitabine. Although the molecular basis of this chemosensitization remains to be fully elucidated, it is consistent with the concept that cytoskeletal organization contributes to stress tolerance and survival under therapeutic pressure. Notably, modulation of Rho GTPase–dependent cytoskeletal programs has previously been shown to influence gemcitabine responsiveness in PDAC, supporting the idea that interference with actin regulatory networks can enhance therapeutic efficacy [19]. These findings suggest that disruption of RHOV-dependent cytoskeletal programs may sensitize PDAC cells to cytotoxic stress, warranting further investigation.

### Limitations and future directions

Several limitations and open questions arise from this work. First, the upstream signals that induce RHOV expression during PDAC progression remain incompletely defined. Our data indicate that RHOV expression is enriched in EMT-associated cellular states; however, the direct transcriptional mechanisms controlling RHOV expression at the promoter level have not yet been defined. Second, while our in vivo studies establish a robust role for RHOV in tumor initiation and metastasis, they were conducted in immunocompromised models, precluding assessment of potential interactions with adaptive immune responses. Additionally, while the intrasplenic injection model is well-suited to study later stages of metastasis such as liver colonization, it does not capture the initial steps of local invasion and intravasation. Future orthotopic studies will complement these findings by assessing RHOV function throughout the full metastatic cascade. Third, while our data demonstrate that RHOV couples EMT-associated transcription to invasive behavior, the current evidence relies largely on transcriptional, morphological, and endpoint invasion assays. Although we performed invasion assays prior to detectable proliferation changes, we cannot fully exclude contributions from altered cell growth. Furthermore, direct assessment of additional structural EMT features, including junction remodeling and polarity changes, will be required to definitively establish RHOV’s role in cytoskeletal EMT execution. Finally, although BRK1 emerges as a key downstream effector of RHOV, it remains to be determined whether additional mechanisms beyond BRK1-dependent WAVE complex regulation contribute to RHOV function in PDAC.

## Conclusions

Taken together, our study identifies RHOV as a previously underappreciated contributor to PDAC aggressiveness that operates by coupling EMT-associated plasticity to cytoskeletal execution through the WAVE–BRK1 axis. By sustaining lamellipodia formation and invasive motility, RHOV supports PDAC cell migration, tumor initiation, and organ colonization in experimental models. Disruption of this pathway impairs invasive behavior, reduces tumor-initiating capacity, and sensitizes PDAC cells to chemotherapy. These findings define a mechanistic link between EMT-associated transcriptional plasticity and invasive actin remodeling and suggest that RHOV-dependent cytoskeletal regulation represents a potential vulnerability in PDAC. It will be interesting to explore whether RHOV plays a similar executional role in other epithelial malignancies.

## Methods

### Culture of primary human PDAC cells

Primary human PDAC cells were derived from resected tumors (SiC020, SiC021, SiC024, SiC038) and circulating tumor cells (CTCs; SiC002, SiC003, SiC005, SiC007, SiC014, SiC610). This study was approved by the ethics committee of Shanghai Jiao Tong University School of Medicine with approval number 2013-0905-70. All study procedures were carried out in accordance with the Declaration of Helsinki. All participants were informed about the purpose of the study, assured of confidentiality, and provided written consent prior to participation. Enrollment was voluntary, and participants could withdraw at any time without any consequences.

Cells were cultured in RPMI-1640 medium supplemented with 10% FBS (#10270106, Gibco) and 1% penicillin/streptomycin (#15140122, Invitrogen) [15]. Authentication was confirmed annually by STR DNA fingerprinting, and all cultures were screened for Mycoplasma every three months by PCR. Only Mycoplasma-negative cells with ≤10 passages were used, and low-passage frozen stocks were periodically refreshed.

### Colony and sphere formation assays

For colony formation, 2×10³ PDAC cells were seeded in 6-well plates and cultured for 14 days. Colonies were fixed and stained with 1% crystal violet and quantified microscopically. For sphere formation, cells were cultured in Ultra-Low Attachment plates (#3471, Corning) at 2,000 cells/mL in DMEM/F12 medium supplemented with 1X B-27™, 20 ng/mL bFGF, penicillin/streptomycin, and amphotericin B (#15290026, Thermo Fisher) for seven days.

### siRNA and plasmid transfection

The siRNAs targeting RHOV and BRK1 are provided in Supplementary Table S1. Cells were seeded in 6-well plates (2×10^5^ per well) and transfected with siRHOV or control siRNA (100 nM final concentration) using Lipofectamine™ 3000 (#L3000015, Thermo Fisher). Cells were harvested 48 h post-transfection for downstream assays (qRT-PCR, Western blotting, or functional assays).

### Western blotting

Cells or tissues were lysed in RIPA buffer (#89900, Thermo Fisher) with protease inhibitors (#11873580001, Sigma). Protein extracts were separated on 15% Bis-Tris gels (#M00719, GenScript) and 4–12% Bis-Tris gels (#M00653, GenScript), transferred to PVDF membranes, blocked, and probed with primary antibodies against WAVE-2 (D2C8, #3659S, Cell Signaling Technology), BRK1 (G-4, #sc-390459, Santa Cruz Biotechnology), ABI1 (D3G6C, #39444 T, Cell Signaling Technology), β-Actin (#AC004, ABclonal) overnight at 4°C. Signal detection was performed using Clarity™ ECL (#1705061, Bio-Rad), and quantification was done with ImageJ.

### Transwell invasion assays

Transwell inserts (8 µm pore size, #3422, Corning) were used. For invasion assays, inserts were pre-coated with 100 µL Growth Factor-Reduced Matrigel (#354230, Corning). PDAC cells were seeded into the upper chambers in serum-free medium, while complete medium served as chemoattractant below. After 24–48 h, cells on the upper surface were removed, and invaded cells were fixed (4% PFA), stained (0.1% crystal violet), imaged (EVOS Imaging System, Thermo Fisher), and counted.

### Wound healing assay

Cells were seeded in 6-well plates at high density. A sterile pipette tip or scratcher was used to make a straight-line scratch in the cell layer, after which detached cells were removed and imaged under microscope (EVOS Imaging System, Thermo Fisher) to monitor cell migration from the edges of the scratch.

### Cell counting kit 8 proliferation assay

Cell proliferation was assessed using the cell counting kit 8 assay (#MA0218, Meilunbio) following the manufacturer’s instructions. PDAC cells were seeded in 96-well plates at a density of 1×10^3^ cells per well and allowed to adhere overnight. At the indicated time points, 10 μL of cell counting kit 8 reagent was added to each well, followed by incubation at 37°C for 1 hour. Absorbance was measured at 450 nm using a BioTek Synergy Neo2 Hybrid multimode microplate reader (BioTek), with signal intensity proportional to the number of viable cells.

### Quantitative real-time PCR (qRT-PCR)

RNA was isolated using SteadyPure Quick RNA Kit (#AG21023, Agbio), and cDNA synthesized using PrimeScript RT Kit (#RR047A, Takara). qRT-PCR was performed with SYBR Green (#FP205, Tiangen) on a QuantStudio™ 6 Pro system. Relative expression was calculated using the 2^–ΔΔCt^ method. Primer sequences are provided in Supplementary Table S1.

### In vivo PDAC models

All animal experiments were conducted in accordance with institutional and national guidelines for the care and use of laboratory animals and were approved by the Institutional Animal Care and Use Committee of Shanghai Jiao Tong University School of Medicine (Project Approval No. A-2020-004).

Six-week-old female BALB/c nude mice were obtained from Shanghai SLAC Laboratory Animal Co., Ltd. (Shanghai, China) and housed under specific pathogen-free (SPF) conditions in individually ventilated cages with controlled temperature (22 ± 2°C), humidity (50–60%), and a 12-h light/dark cycle. Animals had ad libitum access to autoclaved food and water and were monitored daily by trained animal care staff.

The sample size for the animal experiments was determined using a priori power calculations to ensure a statistical power of ≥80% for detecting the anticipated effect size, informed by prior experimental experience with this model. Animals were randomly assigned to experimental groups. Investigators were not blinded during experimentation. For limiting dilution assays, human PDAC cells were resuspended in growth factor-reduced Matrigel™ and injected subcutaneously into the flank at the indicated cell doses in a total volume of 100 µL. Tumor growth was monitored weekly using caliper measurements.

For experimental metastasis assays, PDAC cells resuspended in 50 µL DPBS were injected into the splenic pulp under anesthesia, followed by splenectomy after 2 minutes to prevent primary splenic tumor growth. Liver and lung metastases were assessed 12 weeks after injection.

All surgical procedures were performed under inhalation anesthesia using isoflurane (2–3% for induction, 1.5–2% for maintenance in oxygen). At experimental endpoints or when humane endpoint criteria were reached, mice were euthanized by CO₂ inhalation followed by cervical dislocation, in accordance with AVMA Guidelines for the Euthanasia of Animals. All efforts were made to minimize animal suffering.

### Phalloidin staining

Cells were fixed (4% PFA), permeabilized (0.1% Triton X-100), blocked (5% BSA), incubated with Phalloidin-iFluor 555 Reagent (#ab176756, Abcam) for 1 hour at room temperature. Nuclei were counterstained with DAPI, and imaging was performed on a Zeiss LSM 900 confocal microscope and Operetta (PerkinElmer).

### Co-immunoprecipitation assay

Co-immunoprecipitation was performed using the Pierce™ Co-Immunoprecipitation Kit (#26149, Thermo Fisher) following the manufacturer’s protocol. WAVE2 was used as the bait protein and immunoprecipitated with a WAVE2 antibody (D2C8, #3659S, Cell Signaling Technology). The presence of associated BRK1 (G-4, #sc-390459, Santa Cruz Biotechnology) and ABI1 (D3G6C, #39444 T, Cell Signaling Technology) in the precipitates was assessed by Western blotting. β-Actin (#AC004, ABclonal) served as a control.

### Immunohistochemistry staining

Formalin-fixed, paraffin-embedded (FFPE) tumor tissues were sectioned at 3 μm. Sections were deparaffinized in xylene, rehydrated through graded ethanol, and endogenous peroxidase activity was quenched with 3% hydrogen peroxide in methanol for 10 min. Antigen retrieval was performed in 0.1 M citrate buffer (pH 6.0) by microwave heating for 10 min. Sections were blocked with 3% BSA for 30 min and incubated overnight at 4°C with primary antibodies against Pan-CK (#914204, BioLegend), Ki67 (#GB151499, Servicebio), CD45 (#GB113886, Servicebio), or RHOV (#sc-515072, Santa Cruz Biotechnology). Species-matched secondary antibodies were applied for 50 min at room temperature. Signal was developed using DAB, followed by hematoxylin counterstaining for 2 min. Sections were dehydrated, cleared in xylene, mounted with mounting medium (#G1404, Servicebio), and imaged by light microscopy.

### Lentivirus production and transduction

The short hairpin RNA sequences targeting human RHOV and the single-guide RNA sequence targeting human RHOV are provided in Supplementary Table S1. Vectors for short hairpin RNA of RHOV [RHOV shRNA in pLKO.1-Puro], knockout of *RHOV* [RHOV sgRNA in LentiCRISPR-Puro] and overexpression of *BRK1* [BRK1-HA in pECMV-MCS-3xFLAG-SV40-Puro] were purchased from QEgene. Third-generation lentiviruses were generated in 293T cells (ATCC CRL-3216) using the respective lentiviral backbone, psPAX2 packaging plasmid (Creative Biogene, cat. #OVT2839), and pMD2.g (Creative Biogene, cat. #fgOVT2792) with polyethylenimine transfection reagent (Polysciences, cat. #23966-1). The viral particles were incubated with the target cells for 48 hours, then removed from the cell culture, and antibiotic selection was initiated 24 hours later. The knockdown or overexpression efficiency was validated using qPCR or Western blotting.

### Sanger sequencing

DNA from single clones was extracted using the Genomic DNA Extraction Kit (#DP304, Tiangen). The edited DNA sequences were amplified through PCR by Platinum II Hot-Start Green PCR Master Mix (2X, # 14001-013, Thermo Fisher). The primers for PCR are provided in Supplementary Table 1.

### RNA sequencing and bioinformatics

RNA-seq libraries were prepared from siRHOV-treated or control PDAC cells and sequenced on an Illumina NovaSeq 6000 platform. Differentially expressed genes were identified using DESeq2 with |log₂FC | ≥1 and *p* ≤ 0.05. Gene Ontology (GO), KEGG, and GSEA analyses were performed using DAVID and GSEA software. RNA-seq data are deposited in the NCBI SRA under accession PRJNA1243292.

### Statistical analysis

Data are presented as mean ± SD, unless otherwise stated. Normality was assessed using the Shapiro–Wilk test. Parametric tests (Student’s t-test or one-way ANOVA) were applied for normally distributed data, whereas non-parametric tests (Mann–Whitney U test or Kruskal–Wallis test) were used otherwise. Survival curves were compared using the log-rank test. Sample size was determined by a priori power analysis (power ≥ 0.8) based on the expected effect size using PASS software and was informed by prior experimental experience and technical replication requirements. A two-sided *P* value < 0.05 was considered statistically significant. Statistical analyses were performed using GraphPad Prism (v10.0) or SPSS (v26.0).

### Ethics and data availability

All animal experiments complied with institutional and national guidelines for the care and use of laboratory animals. Human sample collection adhered to the Declaration of Helsinki and was approved under approval number 2013-0905-70.

Public RNA-seq data for PDAC were retrieved from TCGA and GEO (GSE71729, GSE42952, GSE19279) via GEPIA2. *RHOV* bulk RNA-seq data are available under PRJNA1243292. All other data supporting the findings are available upon request from the corresponding author.

## Supplementary information

Supplementary figures and table

Western blot - raw files

## Data Availability

RNA-sequencing data generated in this study have been deposited in the NCBI Sequence Read Archive (SRA) under accession number PRJNA1243292. Publicly available datasets were obtained from TCGA and GEO (GSE71729, GSE42952, GSE19279). All other data supporting the findings of this study are available from the corresponding authors upon reasonable request.
