# Supplementary material for: RHOV couples EMT-associated plasticity to cytoskeletal execution of invasion and metastasis
Source: Cell Death Discov. 2026 May 7;12:285. doi: 10.1038/s41420-026-03137-4 (PMC13319111; doi:10.1038/s41420-026-03137-4)
Supplement: Supplementary file 1 — Supplementary figures and table [file 41420_2026_3137_MOESM1_ESM.pdf]

Figure S1 – RHOV is overexpressed and predicts poor outcome in PDAC

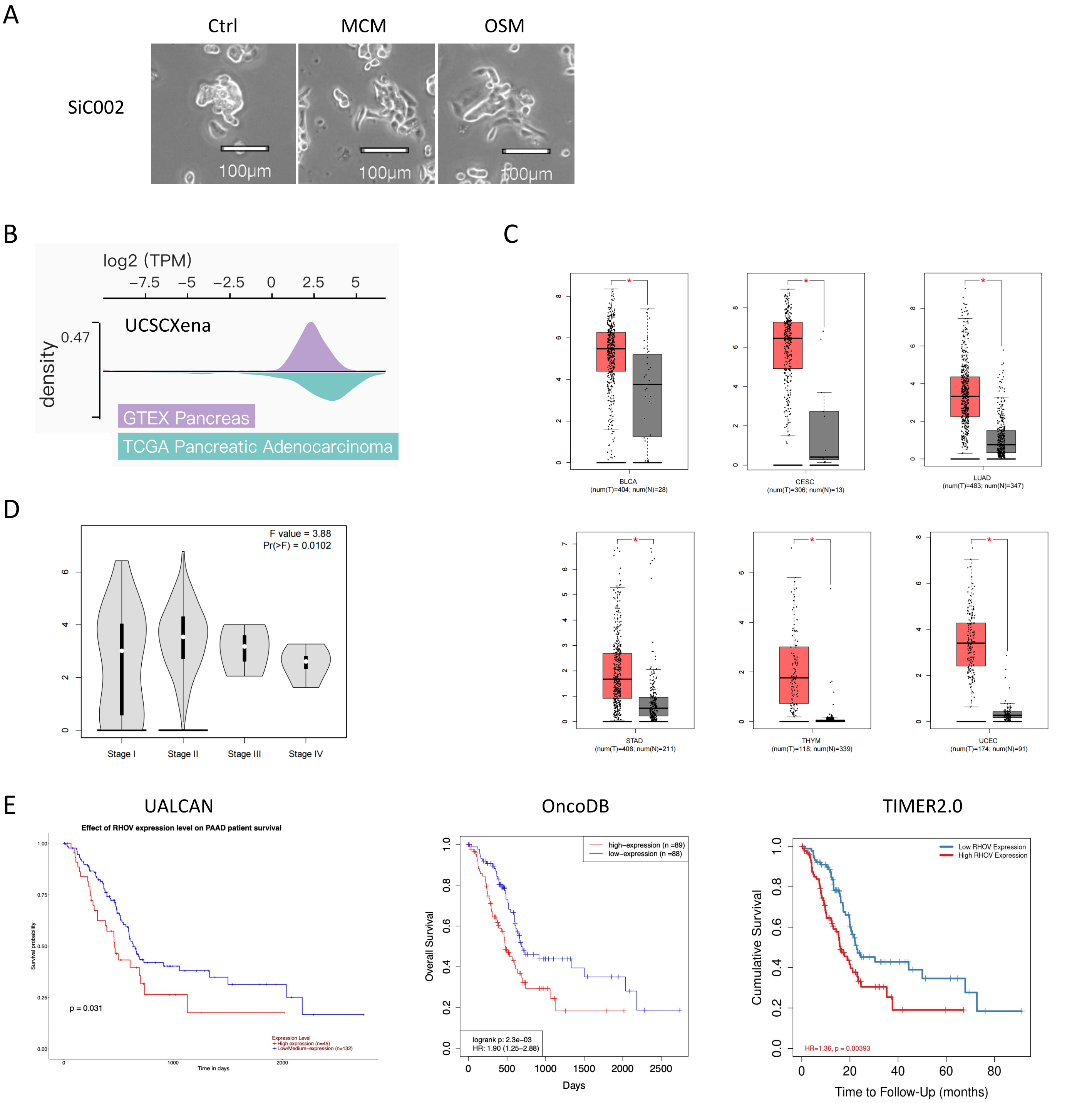

(**A**) Phase-contrast images of SiC002 cells under control (Ctrl), macrophage-conditioned medium (MCM), and oncostatin M (OSM) conditions. (**B**) Density plot of *RHOV* expression (log2 TPM) in TCGA pancreatic adenocarcinoma and GTEx normal pancreas generated using UCSC Xena. (**C**) Box plots of *RHOV* mRNA expression in tumor tissue compared with corresponding normal tissue across multiple cancer types generated using GEPIA. Each dot represents an independent patient. (**D**) Violin plot of *RHOV* mRNA expression across pathological stages of PDAC (Stage I–IV) using GEPIA. (**E**) Kaplan–Meier survival analyses comparing patients with high and low *RHOV* expression using public databases. Left, UALCAN. Middle, OncoDB. Right, TIMER2.0.

Abbreviations: BLCA, bladder urothelial carcinoma; CESC, cervical squamous cell carcinoma and endocervical adenocarcinoma; LUAD, lung adenocarcinoma; STAD, stomach adenocarcinoma; THYM, thymoma; UCEC, uterine corpus endometrial carcinoma.

Figure S2 – RHOV promotes aggressiveness of human PDAC cells

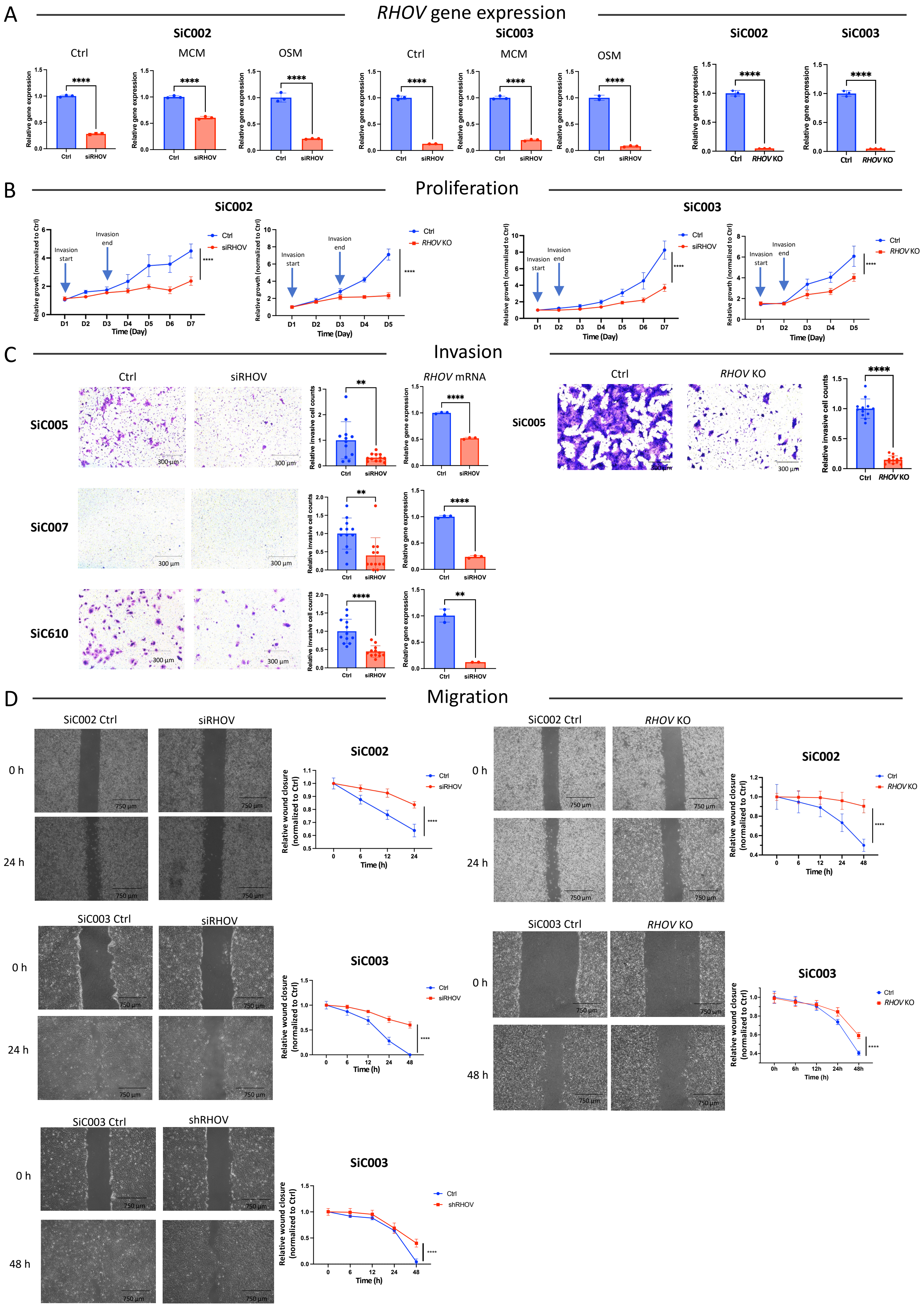

Figure S2 – RHOV promotes aggressiveness of human PDAC cells

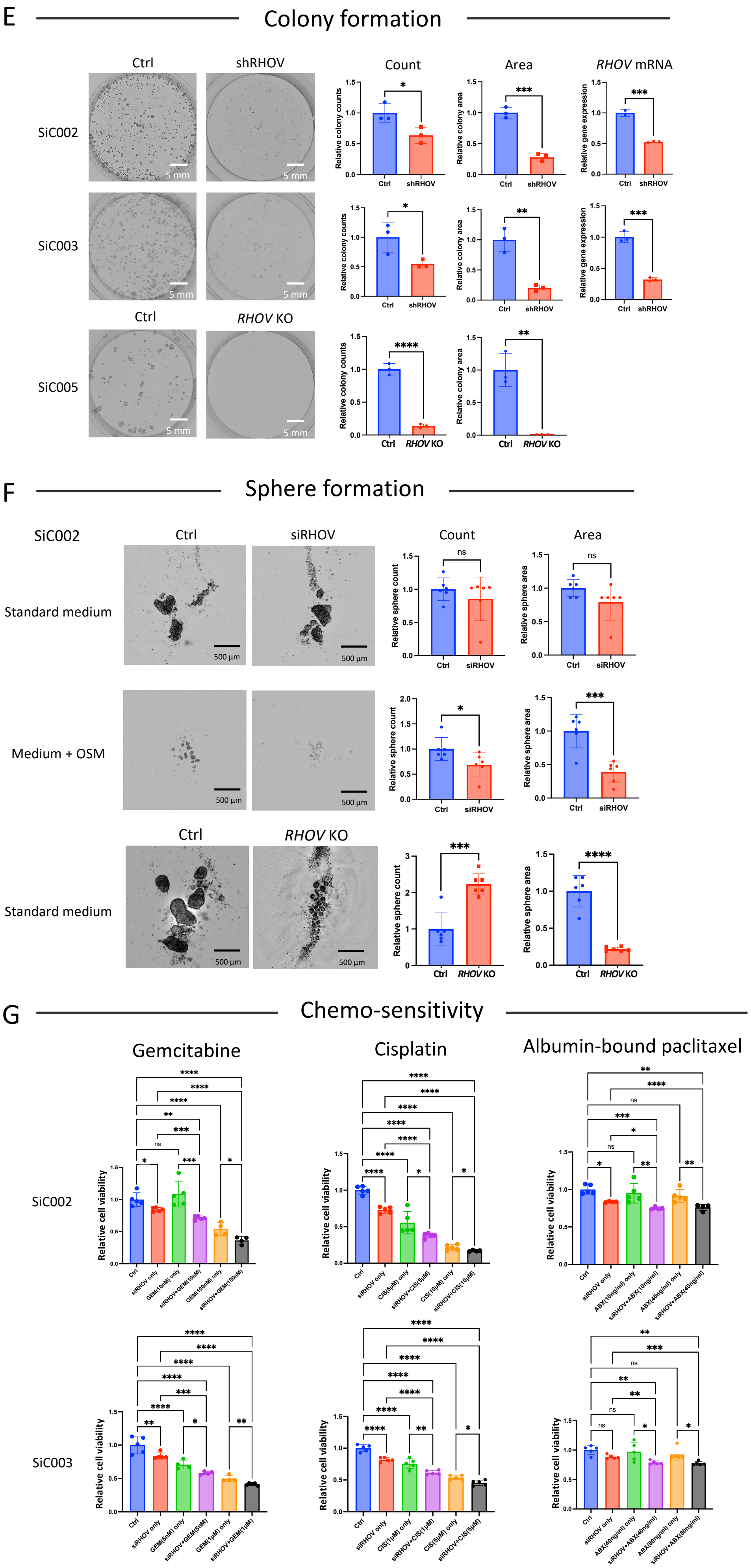

(A) Gene expression. qPCR analysis of RHOV mRNA expression following transient RHOV silencing (siRHOV) in SiC002 and SiC003 cells cultured under standard conditions, MCM, or OSM. Right panels show RHOV expression in RHOV knockout (KO) single-cell clones derived from SiC002 and SiC003 compared with corresponding control cells. Data are based on n = 3 independent qPCR measurements per condition. (B) Cell proliferation curves of SiC002 and SiC003 cells following RHOV knockdown by siRNA (siRHOV) or in RHOV KO single-cell clones, compared with control cells, measured over time (n = 8 independently analyzed wells per time point). (C) Matrigel invasion assays. Representative images and quantification of invasive cells in multiple patient-derived PDAC cultures following RHOV knockdown by siRNA (SiC005, SiC007, SiC610) or RHOV knockout (SiC005 KO clone). (D) Wound healing assays assessing migratory capacity of SiC002 and SiC003 cells following RHOV knockdown by siRNA, shRNA (shRHOV), or RHOV knockout, compared with control cells. Representative images at indicated time points compared with corresponding controls. Each dot represents one independently imaged transwell insert. points and corresponding quantification are shown (n = 5 independently analyzed wounds per time point). (E) Colony formation assays in SiC002 and SiC003 following stable RHOV suppression by shRNA or RHOV knockout (SiC005 KO), with quantification of colony number and colony area relative to controls. Each dot represents one independently analyzed well. (F) Sphere formation. Tumor sphere formation assays in SiC002 cells following RHOV knockdown by siRNA under standard conditions or OSM treatment, or RHOV knockout, compared with control cells. Representative images and quantification of sphere number and sphere area are shown. Each dot represents one independently analyzed culture well. (G) Relative cell viability of SiC002 and SiC003 PDAC cells following RHOV knockdown (siRHOV) and treatment with chemotherapeutic agents at the indicated concentrations. Cells were treated with gemcitabine (GEM), cisplatin (CIS), or albumin-bound paclitaxel (ABX) alone or in combination with siRHOV. Each dot represents one independently analyzed culture well. Statistical significance is indicated as * $p < 0.05$, ** $p < 0.01$, *** $p < 0.001$, and **** $p<0.0001$. Data are presented as mean $\pm$ SD.

Figure S3 – RHOV enhances tumor initiation and metastasis *in vivo* .

Subcutaneous injection of human PDAC cells

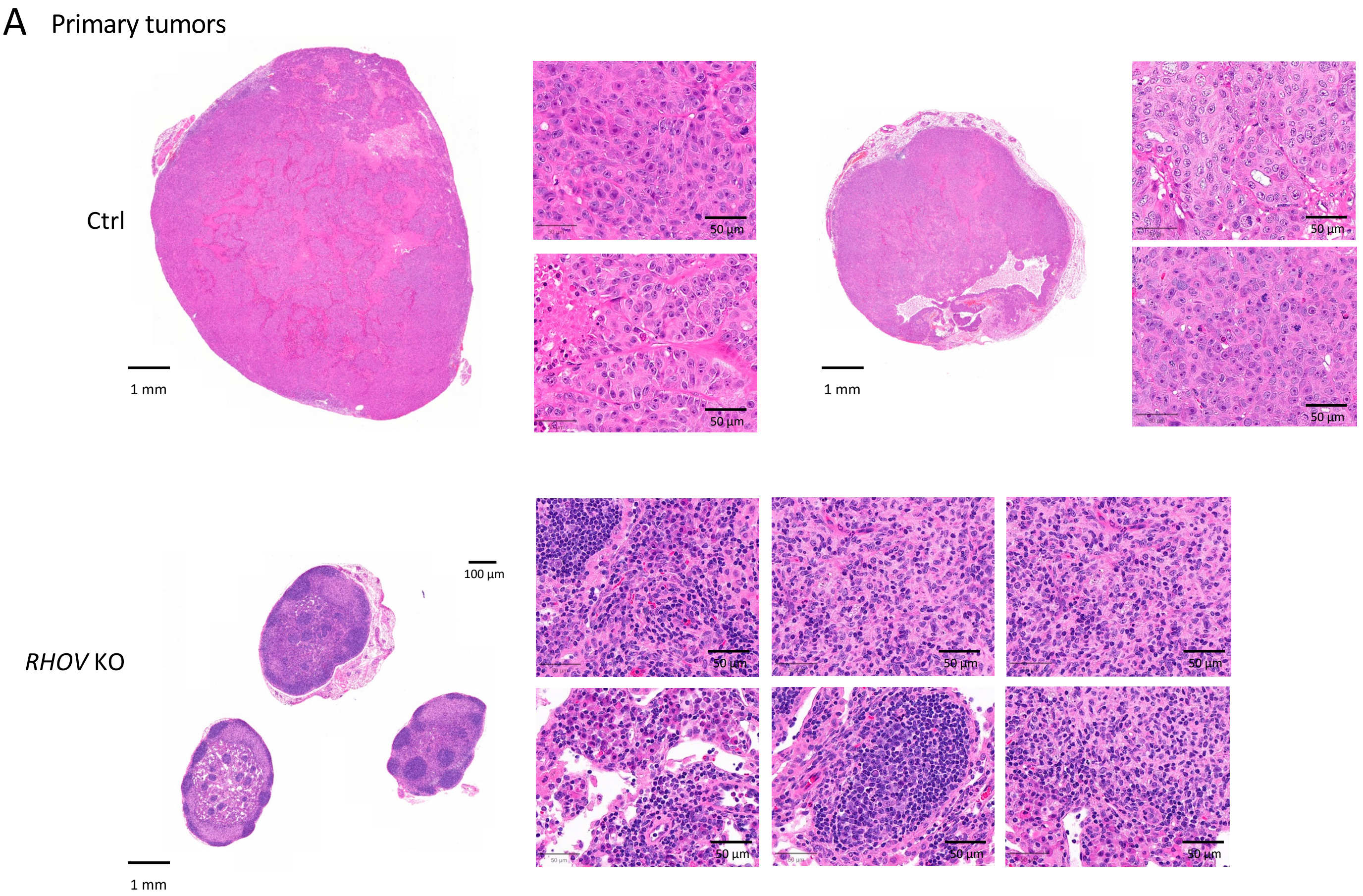

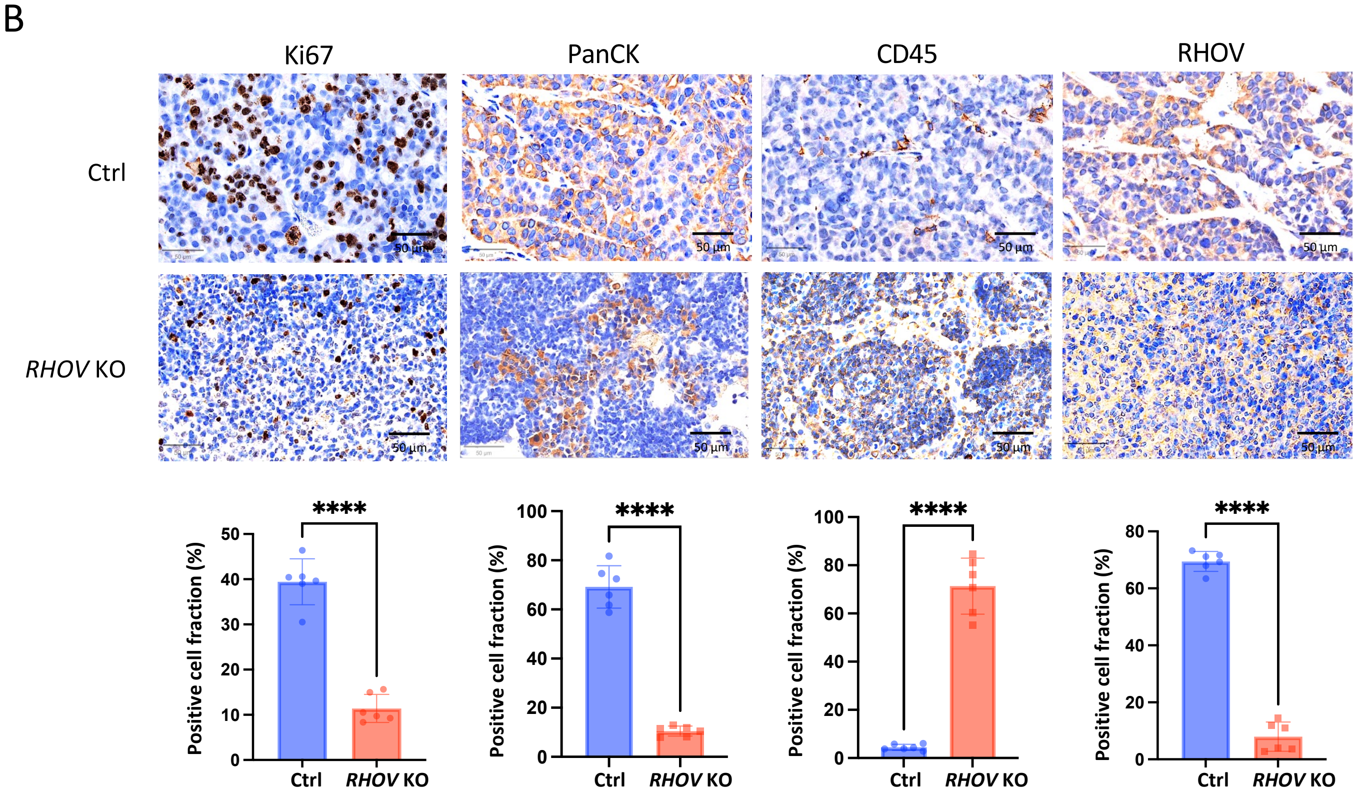

Figure S3 – RHOV enhances tumor initiation and metastasis *in vivo* .

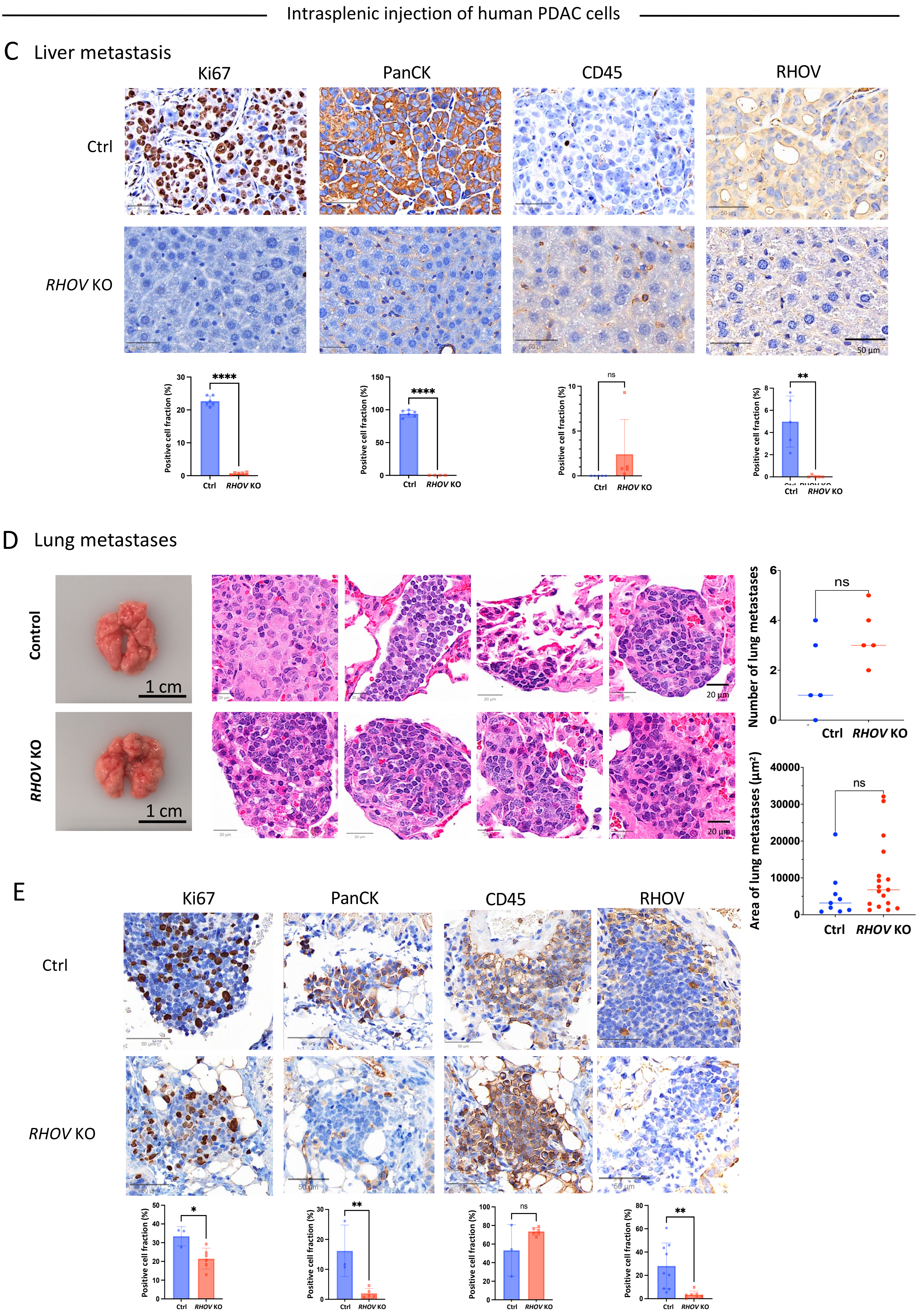

(A) Representative H&E-stained sections of subcutaneous tumors derived from control or *RHOV* knockout SiC002 cells following subcutaneous injection. Top, control tumors of varying sizes. Bottom, *RHOV* KO tumors. Insets show higher-magnification views of tumor histology. (B) Immunohistochemical staining of subcutaneous tumors for Ki-67, PanCK, CD45, and RHOV in control and *RHOV* KO groups. Quantification shown below. (C) Liver metastases following intrasplenic injection of control or *RHOV* KO SiC002 cells. Representative immunohistochemical staining for Ki-67, PanCK, CD45, and RHOV in metastatic lesions. Quantification shown below. (D) Lung metastases following intrasplenic injection of control or *RHOV* KO SiC002 cells. Representative gross images and H&E-stained sections are shown. Quantification shown on the right (n = 5 mice per group). (E) Immunohistochemical staining of lung metastatic lesions for Ki-67, PanCK, CD45, and RHOV in control and *RHOV* KO groups. Quantification shown below. Statistical significance is indicated as * $p < 0.05$, ** $p < 0.01$, and **** $p < 0.0001$. Data are presented as mean $\pm$ SD.

Figure S4 – RHOV regulates lamellipodia formation through the WAVE regulatory complex

A *RHOV* knockdown by siRHOV

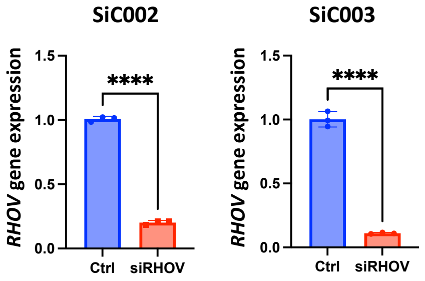

B

Gene ID	Gene Symbol	log2FC (002siRHOV/002Ctrl)	Significance (002DEG)	log2FC (003siRHOV/003Ctrl)	Significance (003DEG)
100533467	BIVM-ERCC5	-11.9683	<0.0001	-1.0872	0.0001
54793	KCTD9	-2.0867	<0.0001	-1.8853	<0.0001
55845	BRK1	-2.0597	<0.0001	-2.2236	<0.0001
307	ANXA4	-1.7764	<0.0001	-1.3795	<0.0001
595	CCND1	-1.7270	<0.0001	-1.9757	<0.0001
28962	OSTM1	-1.5013	<0.0001	-1.5974	<0.0001
5832	ALDH18A1	-1.4688	<0.0001	-1.5155	<0.0001
100526832	PHOSPHO2-KLHL23	-1.3642	<0.0001	-1.9835	<0.0001
26973	CHORDC1	-1.3085	<0.0001	-1.1036	<0.0001
10797	MTHFD2	-1.2906	<0.0001	-1.3900	<0.0001
25923	ATL3	-1.2749	<0.0001	-1.3188	<0.0001
84275	SLC25A33	-1.2748	<0.0001	-1.2257	<0.0001
8263	F8A1	-1.2712	<0.0001	-1.2064	<0.0001
9937	DCLRE1A	-1.2605	<0.0001	-1.6507	<0.0001
8317	CDC7	-1.2486	<0.0001	-1.6934	<0.0001
4791	NFKB2	-1.2437	<0.0001	-1.9006	<0.0001
54830	NUP62CL	-1.1575	<0.0001	-1.5272	<0.0001
171177	RHOV	-1.1535	<0.0001	-2.5041	<0.0001
153768	PRELID2	-1.1405	<0.0001	-1.1439	<0.0001
6996	TDG	-1.1381	<0.0001	-1.1901	<0.0001
55038	CDCA4	-1.1073	<0.0001	-1.6268	<0.0001
4245	MGAT1	-1.1065	<0.0001	-1.2183	<0.0001
116028	RMI2	-1.0973	<0.0001	-1.3384	<0.0001
11277	TREX1	-1.0900	0.0003	-2.0821	0.0005
79847	MFSD13A	-1.0663	0.0001	-1.2469	0.0002
899	CCNF	-1.0552	<0.0001	-1.4838	<0.0001
9319	TRIP13	-1.0489	<0.0001	-1.7833	<0.0001
3029	HAGH	-1.0336	<0.0001	-1.6280	<0.0001
56650	CLDND1	-1.0217	<0.0001	-1.4738	<0.0001
222068	TMED4	-1.0084	<0.0001	-1.0076	<0.0001
3017	H2BC5	1.0000	<0.0001	1.8931	<0.0001
3554	IL1R1	1.0120	<0.0001	1.1288	<0.0001
113189	CHST14	1.0220	<0.0001	1.0286	<0.0001
23193	GANAB	1.0347	<0.0001	1.0442	<0.0001
144717	PHETA1	1.0772	<0.0001	1.3870	<0.0001
11054	OGFR	1.1079	<0.0001	1.3005	<0.0001
7171	TPM4	1.1392	<0.0001	1.1821	<0.0001
3491	CCN1	1.2022	<0.0001	1.2877	<0.0001
10537	UBD	1.3084	<0.0001	-1.0194	<0.0001
8537	BCAS1	1.4150	<0.0001	1.1715	<0.0001
84952	CGNL1	1.5166	<0.0001	1.3866	<0.0001
284040	CDRT4	1.6976	<0.0001	12.2491	<0.0001
90317	ZNF616	1.7111	<0.0001	1.4980	<0.0001
22927	HABP4	1.7270	<0.0001	1.6853	<0.0001
415117	STX19	1.8480	0.0004	1.0066	<0.0001
728689	EIF3CL	12.7895	<0.0001	11.0614	<0.0001

Figure S4 – RHOV regulates lamellipodia formation through the WAVE regulatory complex

C Pathway enrichment of 47 overlapping DEGs

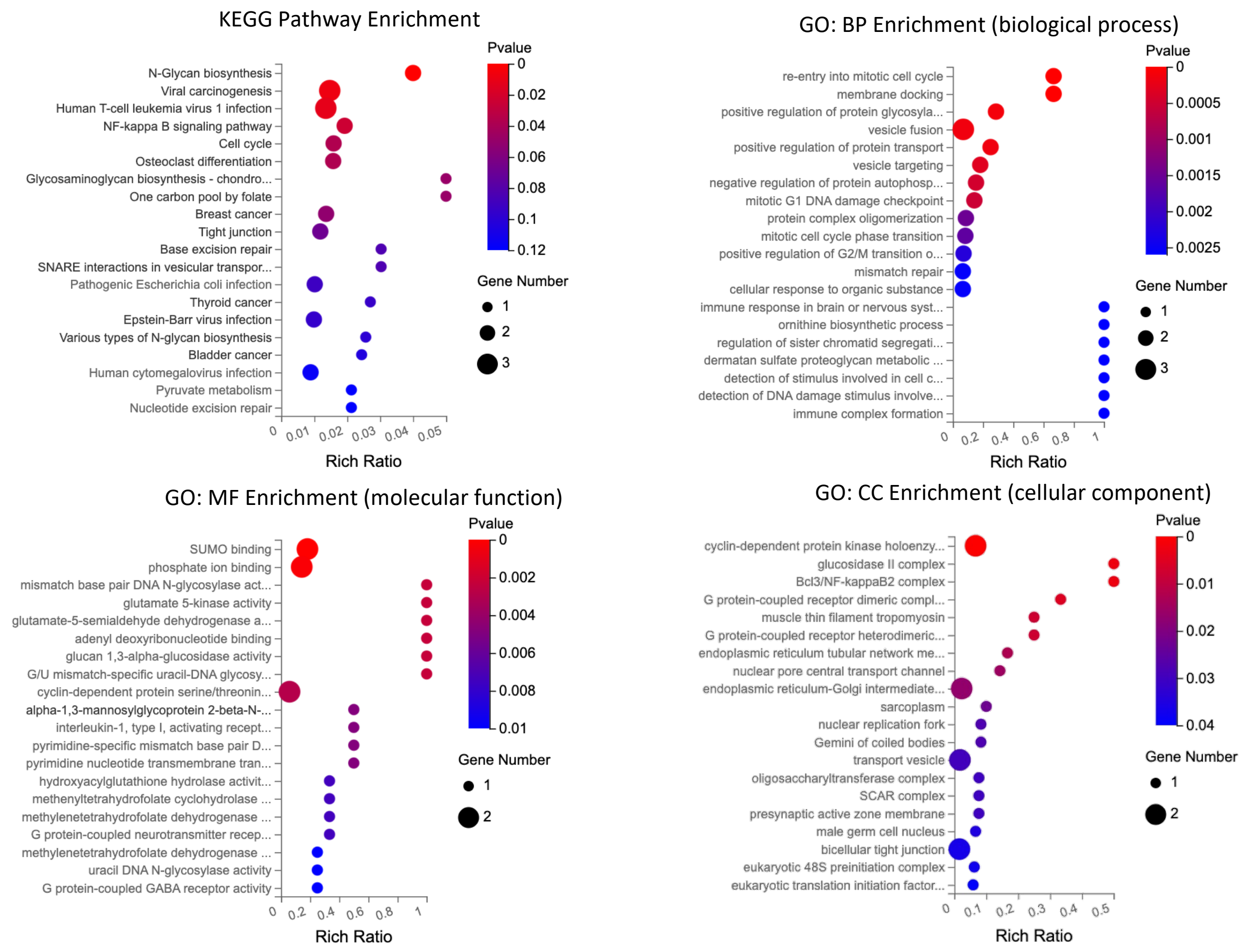

D Selected WRC genes after RHOV knockdown (RNA-seq)

Gene ID	Gene Symbol	log2FC (002siRHOV/002Ctrl)	Significance (002)	log2FC (003siRHOV/003Ctrl)	Significance (003)
8936	WAVE1	0.0855	0.4372	-0.0698	0.8225
10163	WAVE2	0.0945	0.0001	0.0133	0.7106
10810	WAVE3	–	–	-0.1478	0.3958
10006	ABI1	0.2176	0.0007	0.2203	<0.0001
10152	ABI2	-0.1692	0.0703	-0.2147	<0.0001
10787	NCKAP1	0.0413	0.0902	0.1209	<0.0001
23191	CYFIP1	0.1809	<0.0001	0.1025	0.0148
26999	CYFIP2	0.6599	0.0009	-0.5850	0.7924
55845	BRK1	-2.0597	<0.0001	-2.2236	<0.0001

E Selected WRC genes after RHOV knockdown (qPCR)

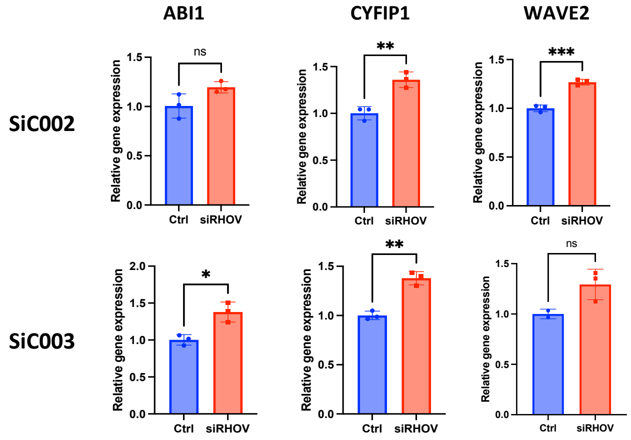

(**A**) qPCR analysis of *RHOV* expression following siRHOV treatment in SiC002 and SiC003 cells (n = 3 independent qPCR measurements per condition). (**B**) Table listing the 47 overlapping differentially expressed genes shared between SiC002 and SiC003 cells after *RHOV* knockdown, including log2 fold change and statistical significance values. (**C**) KEGG pathway and Gene Ontology (GO) enrichment analyses (biological process, molecular function, and cellular component) of the 47 overlapping differentially expressed genes identified after *RHOV* knockdown in SiC002 and SiC003 cells. (**D**) RNA-seq analysis of selected WRC genes in SiC002 and SiC003 cells following *RHOV* knockdown. (**E**) qPCR validation of selected WRC genes in SiC002 and SiC003 cells following *RHOV* knockdown (n = 3 independent qPCR measurements per condition). Statistical significance is indicated as * $p < 0.05$, ** $p < 0.01$, *** $p < 0.001$, and **** $p < 0.0001$. Data are presented as mean ± SD.

Figure S5 – RHOV drives PDAC invasiveness through BRK1

A *BRK1* gene expression

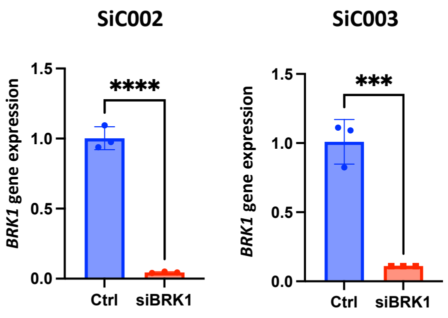

B *RHOV* gene expression

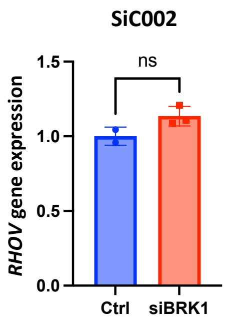

C Effect of *BRK1* knockdown on PDAC proliferation

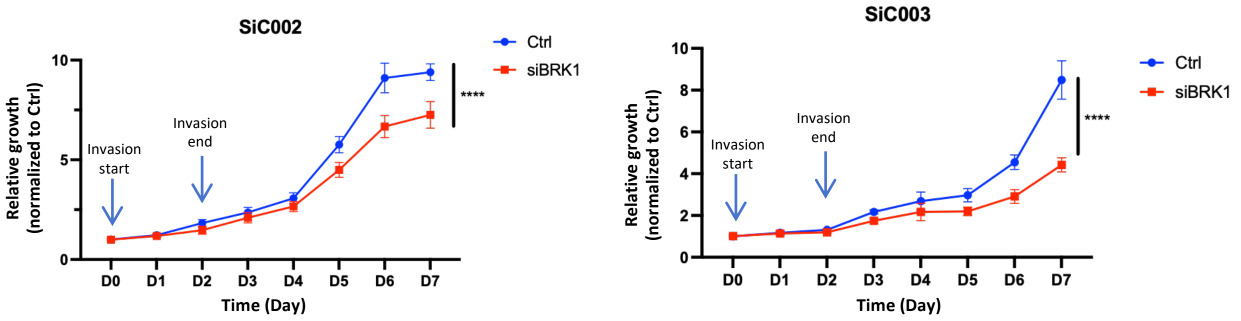

D *BRK1* and *RHOV* gene expression in rescue experiments

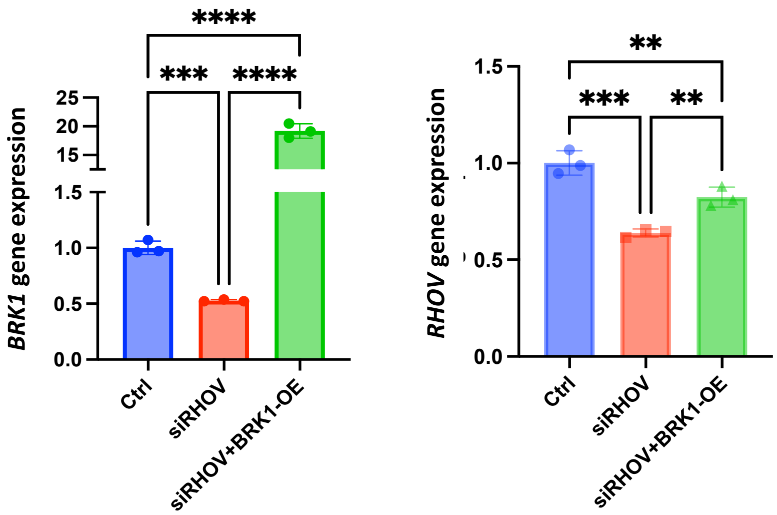

(**A**) qPCR confirming efficient *BRK1* mRNA knockdown after siBRK1 transfection, relative to scrambled control, in SiC002 and SiC003 cells (n = 3 independent qPCR measurements per condition). (**B**) qPCR analysis of *RHOV* mRNA expression in SiC002 cells following *BRK1* knockdown, relative to scrambled control (n = 3 independent qPCR measurements per condition). (**C**) Relative proliferation of SiC002 and SiC003 cells after *BRK1* knockdown over a 7-day period (scrambled control vs siBRK1). Arrows indicate the time window corresponding to the invasion assays shown in Figure 5C. (**D**) qPCR analysis of *BRK1* and *RHOV* mRNA expression in SiC002 cells following *RHOV* inhibition in the presence or absence of *BRK1* overexpression, relative to scrambled control (n = 3 independent qPCR measurements per condition). Statistical significance is indicated as ** $p < 0.01$, *** $p < 0.001$, and **** $p < 0.0001$. Data are presented as mean $\pm$ SD.

Supplementary Table 1

Primers for qPCR	
RHOV-F	CCTCATCGTCAGCTACACCTG
RHOV -R	GAACGAAGTCGGTCAAAATCCT
BRK1-F	GTGCAGCGGGAGATTCACC
BRK1-R	AACGACAAGACATATCGAACGAG
ABI1-F	ACCAGTCCTGCTAGGCTTG
ABI1-R	ACTGTTTTCTCGACTTCCACTTC
WAVE2-F	CTCGGGTAAGCTCCCTTGC
WAVE2-R	GCTTTTCGGGTGTTGATTCCTT
CYFIP1-F	TCCCCATTGAGATGTCGATGC
CYFIP1-R	ACTGCTTGTTGAACCTGGTGA

Primer for Sanger sequence	
RHOV DNA-F	TGAGAAATGGATTCCATGTGTCGG
RHOV DNA-R	ACGAAGCAGAAGAACTTCTTCCAG

siRNA sequence	
RHOV #1	CAGCGGGACAGGAGGAUUUTT
RHOV #2	AAAUCCUCCUGUCCCGCUGTT
BRK1 #1	CGAUAUGUCUUGUCGUUCATT
BRK1 #2	UGAACGACAAGACAUAUCGTT

shRNA sequence	
**RHOV #1**	CGGCTGGAGAAGAAACTGAAT
**RHOV #2**	GAAGTATTTGACTCGGCTATT

sgRNA sequence	
**RHOV #1**	GAAGCACGCCAGGAAGACAT
**RHOV #2**	GTTGACATCGTCCCTCAGGT
