# Supplementary figures and images for: RHOV couples EMT-associated plasticity to cytoskeletal execution of invasion and metastasis
Source: Cell Death Discov. 2026 May 7;12:285. doi: 10.1038/s41420-026-03137-4 (PMC13319111; doi:10.1038/s41420-026-03137-4)

Western blot raw files

Figure 4D

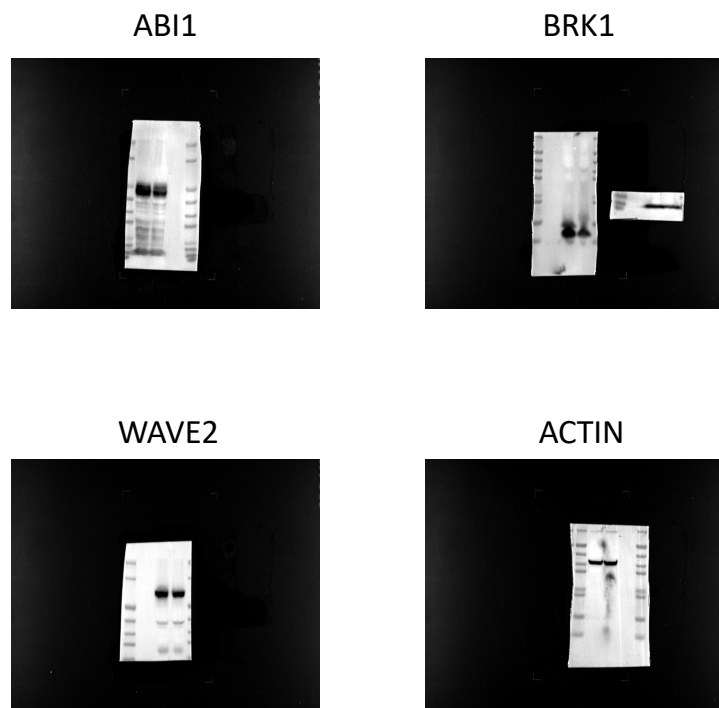

Figure 4E

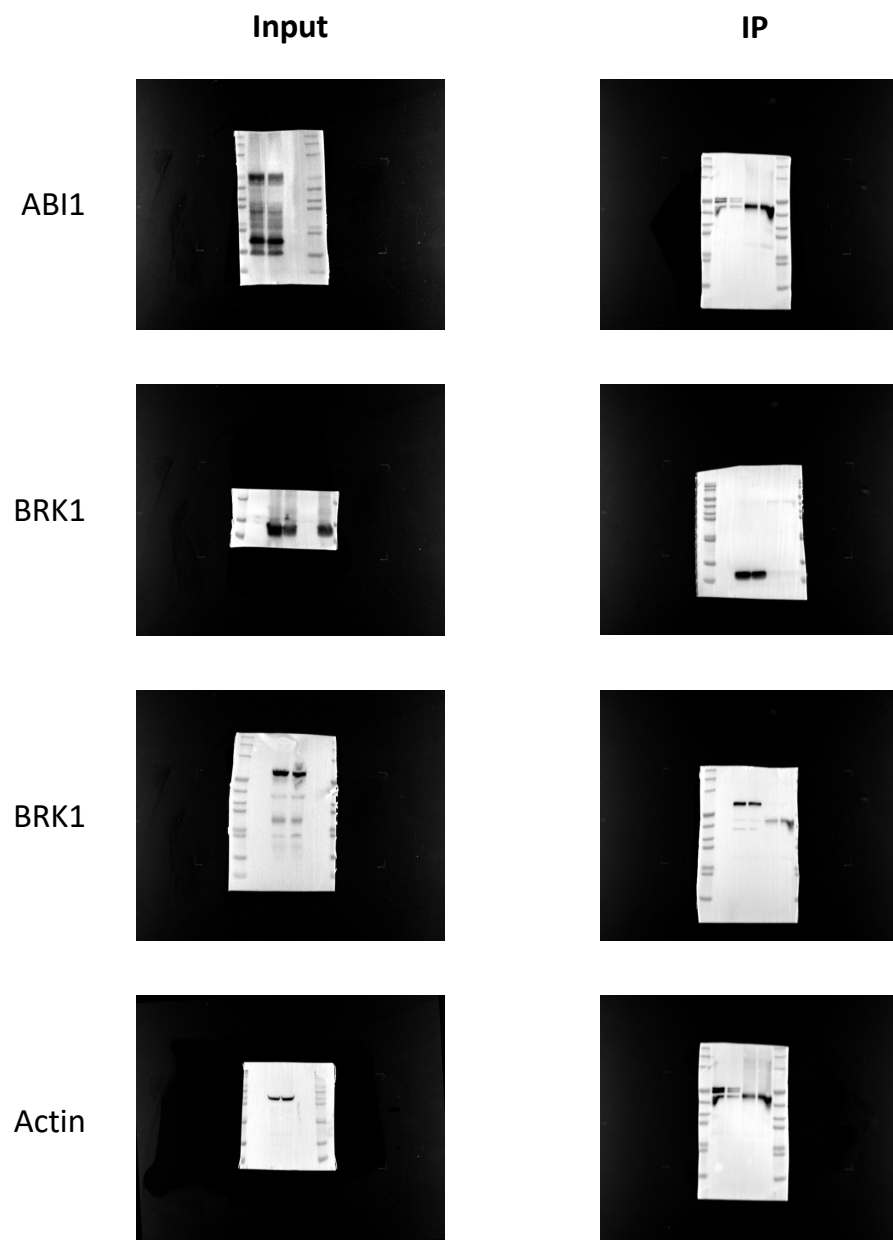

Supplement: Supplementary file 2 — Western blot - raw files [file 41420_2026_3137_MOESM2_ESM.pdf]
